# A drift diffusion model analysis of age-related impact on multisensory decision-making processes

**DOI:** 10.1038/s41598-024-65549-5

**Published:** 2024-06-28

**Authors:** Joshua Bolam, Jessica A. Diaz, Mark Andrews, Rachel O. Coats, Marios G. Philiastides, Sarah L. Astill, Ioannis Delis

**Affiliations:** 1 School of Biomedical Sciences, University of Leeds, West Yorkshire, LS2 9JT UK; 2 School of Social Sciences, Birmingham City University, West Midlands, B15 3HE UK; 3 School of Social Sciences, Nottingham Trent University, Nottinghamshire, NG1 4FQ UK; 4 School of Psychology, University of Leeds, West Yorkshire, LS2 9JT UK; 5 School of Neuroscience and Psychology, University of Glasgow, Lanarkshire, G12 8QB UK; 6 Institute of Neuroscience, Trinity College Dublin, Dublin, D02 PX31 Ireland

**Keywords:** Ageing, Perceptual decision-making, Multisensory decision-making, Audiovisual object categorisation paradigm, Hierarchical drift diffusion model(ling) (HDDM), Human behaviour, Cognitive ageing

## Abstract

Older adults (OAs) are typically slower and/or less accurate in forming perceptual choices relative to younger adults. Despite perceptual deficits, OAs gain from integrating information across senses, yielding multisensory benefits. However, the cognitive processes underlying these seemingly discrepant ageing effects remain unclear. To address this knowledge gap, 212 participants (18–90 years old) performed an online object categorisation paradigm, whereby age-related differences in Reaction Times (RTs) and choice accuracy between audiovisual (AV), visual (V), and auditory (A) conditions could be assessed. Whereas OAs were slower and less accurate across sensory conditions, they exhibited greater RT decreases between AV and V conditions, showing a larger multisensory benefit towards decisional speed. Hierarchical Drift Diffusion Modelling (HDDM) was fitted to participants’ behaviour to probe age-related impacts on the latent multisensory decision formation processes. For OAs, HDDM demonstrated slower evidence accumulation rates across sensory conditions coupled with increased response caution for AV trials of higher difficulty. Notably, for trials of lower difficulty we found multisensory benefits in evidence accumulation that increased with age, but not for trials of higher difficulty, in which increased response caution was instead evident. Together, our findings reconcile age-related impacts on multisensory decision-making, indicating greater multisensory evidence accumulation benefits with age underlying enhanced decisional speed.

## Introduction

When forming rapid decisions, incoming sensory information is often processed across multiple modalities, and then exploited for *multisensory decision-making*^1,2^. Ageing has been demonstrated to affect: (1) *multisensory integration*; integrating sensory information across modalities into unified percepts^3,4^, and (2) *perceptual decision-making*; translating immediately available sensory information into choice behaviours^5,6^. Previous research investigating multisensory integration has demonstrated that older adults exhibit preserved (and to an extent enhanced^7–9^) multisensory response facilitation relative to younger adults^10–12^. This finding accompanies a common observation in perceptual decision-making research: that older adults exhibit larger reaction times (RTs) in speeded paradigms than younger adults^13–15^, suggesting that older adults also react more slowly in accumulating information when forming perceptual decisions.

Given our environment is inherently multisensory, and most of, if not all, speeded paradigms require a rapid decision to be facilitated based on immediately presented stimuli^13,16,17^, it is important to consider multisensory integration an integral component of perceptual decision-making^18^, particularly in ageing research where the reliability of incoming (multi)sensory information can be impacted by variations in task difficulty^13,15,19^. For example, whereas it has been demonstrated that older adults manifest age-related decrements in perceptual decision-making and attentional engagement under higher levels of task difficulty, or reduced perceptual sensitivity^20–22^, they display preserved multisensory benefits from integrating unisensory signals that are less coherent and therefore increasingly difficult to consolidate separately^10,12,23^. By considering the impact of ageing on the interplay between multisensory integration and perceptual decision-making, as well as understanding the modulatory influence of task difficulty within this interplay, we can begin to understand whether multisensory decision-making processes remain preserved or degraded across the adult lifespan.

One suggested approach, from perceptual decision-making research^13–15^, is to use computational modelling. In particular, *sequential sampling modelling approaches*^24–26^ assume that perceptual decisions are formed by stochastically accumulating noisy sensory information until a decision threshold is exceeded, and then dissect the constituent processes underlying choice formation. The *Drift Diffusion Model* (DDM^27–29^), for example, analyses RT distributions and choice accuracy in Two-Alternative Forced-Choice (2AFC) paradigms, and decomposes them into the following latent cognitive components underlying the perceptual decision formation process: (1) the rate of sensory evidence accumulated in the decision process (i.e., *drift rate*), (2) the degree of response caution quantifying the decision criterion (i.e., *decision boundary*), and (3) the duration of processes not attributable to sensory evidence accumulation; such as sensory encoding and motor response latency (i.e., *non-decision time*). Therefore, by decomposing a behavioural dataset of different age groups into DDM parameters, age-related processes that drive changes in decision behaviour can be inferred, benefitting our understanding of the affected processes leading to observed choice outcomes. For example, slower responses, combined with higher choice accuracy, can be attributed to increased response caution, which is captured by the decision boundary parameter^30,31^.

Research applying sequential sampling modelling approaches, most notably the DDM, to probe age-related impacts on *unisensory* perceptual decision-making behaviour has provided valuable insights into the key computations affected^13–15,21,32–38^. To our knowledge, however, few studies have applied such approaches in order to probe age-related impacts on *multisensory* decision-making processes. One identified study, from Jones et al.^19^, modelled the effects of ageing on multisensory decision-making for audiovisual spatial localisation. They demonstrated similar patterns of audiovisual binding tendency between younger and older adults in localization and common-source judgements, albeit disproportionally longer RTs when localising strongly incongruent audiovisual signals. Behavioural modelling inferred that older adults sacrificed response speed to compensate for encoding noisier sensory representations (particularly for auditory signals), and thus set higher decision thresholds when accumulating evidence, to preserve the choice performance outcomes. Accordingly, this study typifies why sequential sampling modelling applications can provide novel insights into the distinct computations impacted within multisensory decision-making performance.

In the present study, we coupled single-trial measurements of multisensory decision-making behaviour, i.e., RTs and choice accuracy, recorded from an internet-based (i.e., online) variant of an audiovisual object categorisation paradigm^2^, with *Hierarchical Drift Diffusion Modelling* (HDDM^39^) to address the aforementioned knowledge gap. By utilising this experimental paradigm, we could examine the extent to which ageing influences multisensory integration within perceptual decision formation. Specifically, we could observe whether consolidating audiovisual information improves the ability to form perceptual decisions compared to auditory or visual information alone. In addition, we could manipulate the coherence of multisensory and unisensory stimulus presentations to further address the understudied influence of task difficulty on object categorisations. HDDM then permitted us a mechanistic insight into the psychologically meaningful latent parameters affected by ageing; not otherwise accessible with standard RT/choice accuracy statistical analyses. Thus, we could differentiate age-related changes in rates of sensory evidence accumulation uptake (i.e., *drift rates*), response caution in decision threshold policies (i.e., *decision boundary*), and duration of non-decisional processing (i.e., *non-decision time*). As a result, we could (a) assess the effects of ageing on the behavioural indices of multisensory decision-making, and (b) dissect the constituent processes underlying identified subsequent age-related modulations, allowing us to probe the internal cognitive mechanisms that are either preserved or degraded in older adults.

## Results

**Figure 1:**
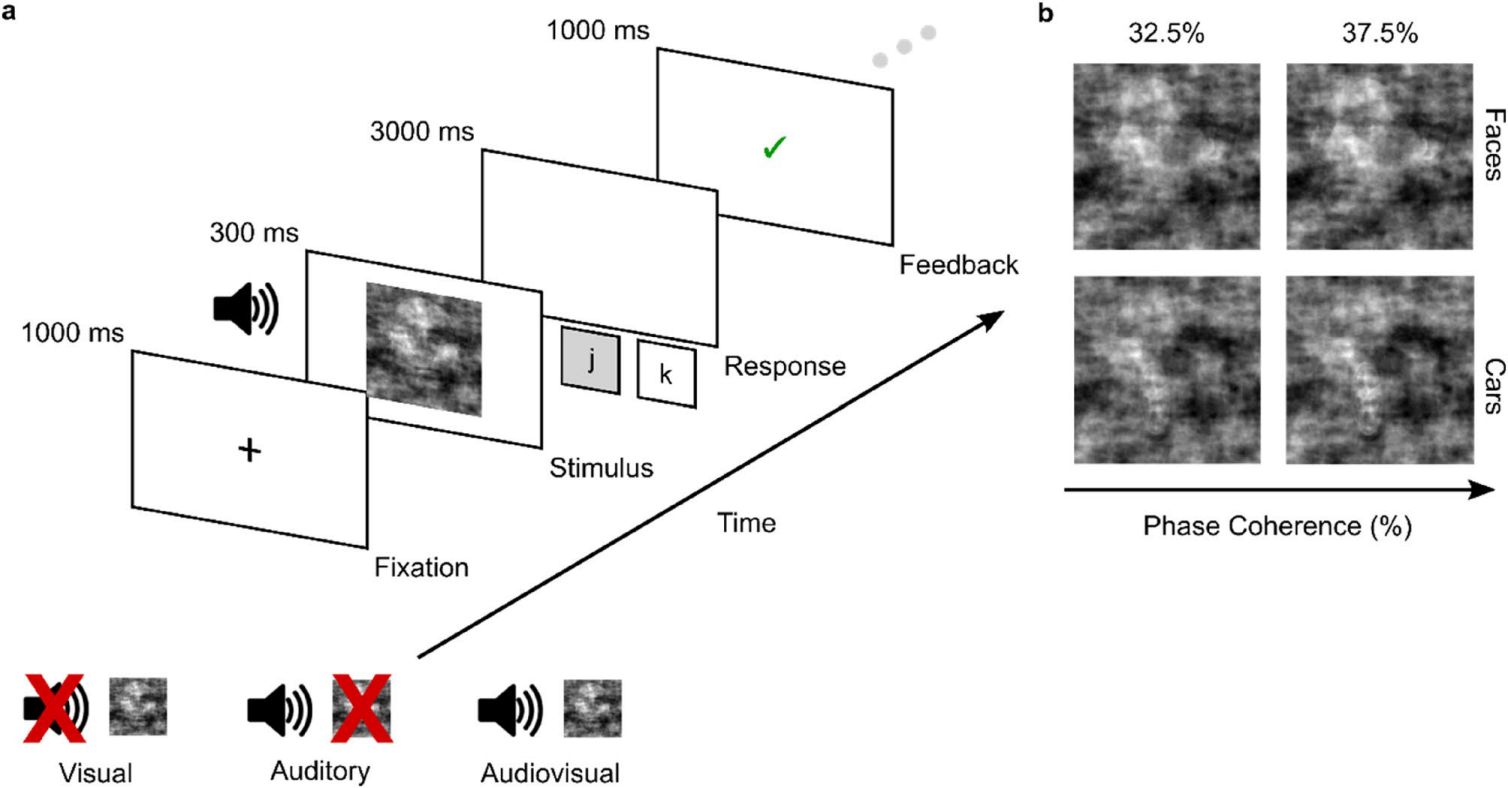
Experimental paradigm. (**a**) Schematic representation of the experimental paradigm on a single-trial basis. Participants were instructed to categorise noisy representations of faces and cars. First, a fixation cross was presented centrally on-screen for 1000 ms. Then, a brief stimulus, which was either an image (Visual, *right*), a sound (Auditory, *middle*), or a simultaneous image-sound pairing (Audiovisual, *right*), was presented for 300 ms, and followed by a delay period of a maximum of 3000 ms during which participants were instructed to indicate their response with a keyboard button press (*j* or *k* keyboard button presses for face/car categorisations respectively). Following their response, feedback was then presented for 1000 ms (a green tick or a red cross, for a correct or incorrect response, respectively), which preceded an inter-stimulus interval (ISI) of 500 ms (illustrated by the ellipsis). (**b**) Sample face (t*op*) and car (*bottom*) images at the two levels of stimulus phase coherence used in the experimental paradigm (Low Coherence: 32.5%; High Coherence: 37.5%).

Participants (N = 212; age range = 18.08–86.83 years) completed an online variant of the audiovisual face-versus-car object categorisation paradigm^2,40^ (Fig. 1a) using the Gorilla Experiment Builder platform (http://www.gorrila.sc^41,42^). This paradigm instructs participants to categorise, as quickly and as accurately as possible, whether a face or a car is embedded in a series of images, sounds, or simultaneously presented images and sounds, with RTs and choice accuracy (binary correct/incorrect responses) collected as single-trial measurements of perceptual decision-making performance. Generalised Linear Mixed-Effects Models (GLMMs) and likelihood-ratio (χ^2^) model comparisons were used to analyse choice accuracy and RTs (using *binomial* logit and *gamma* models respectively) as a function of sensory condition (Visual: V, Auditory: A, Audiovisual: AV trials), stimulus phase coherence (High Coherence: HC/Low Coherence: LC levels respectively, Fig. 1b), and a chronological, or *continuous*, age predictor, as well as subsequent two-way and three-way interactions (see *Supplementary Materials* S1 and S2 for a full overview of GLMM analyses, including with a *categorical* age predictor—see *Supplementary Materials* S3, S4, and S5).

### Behavioural results

**Figure 2:**
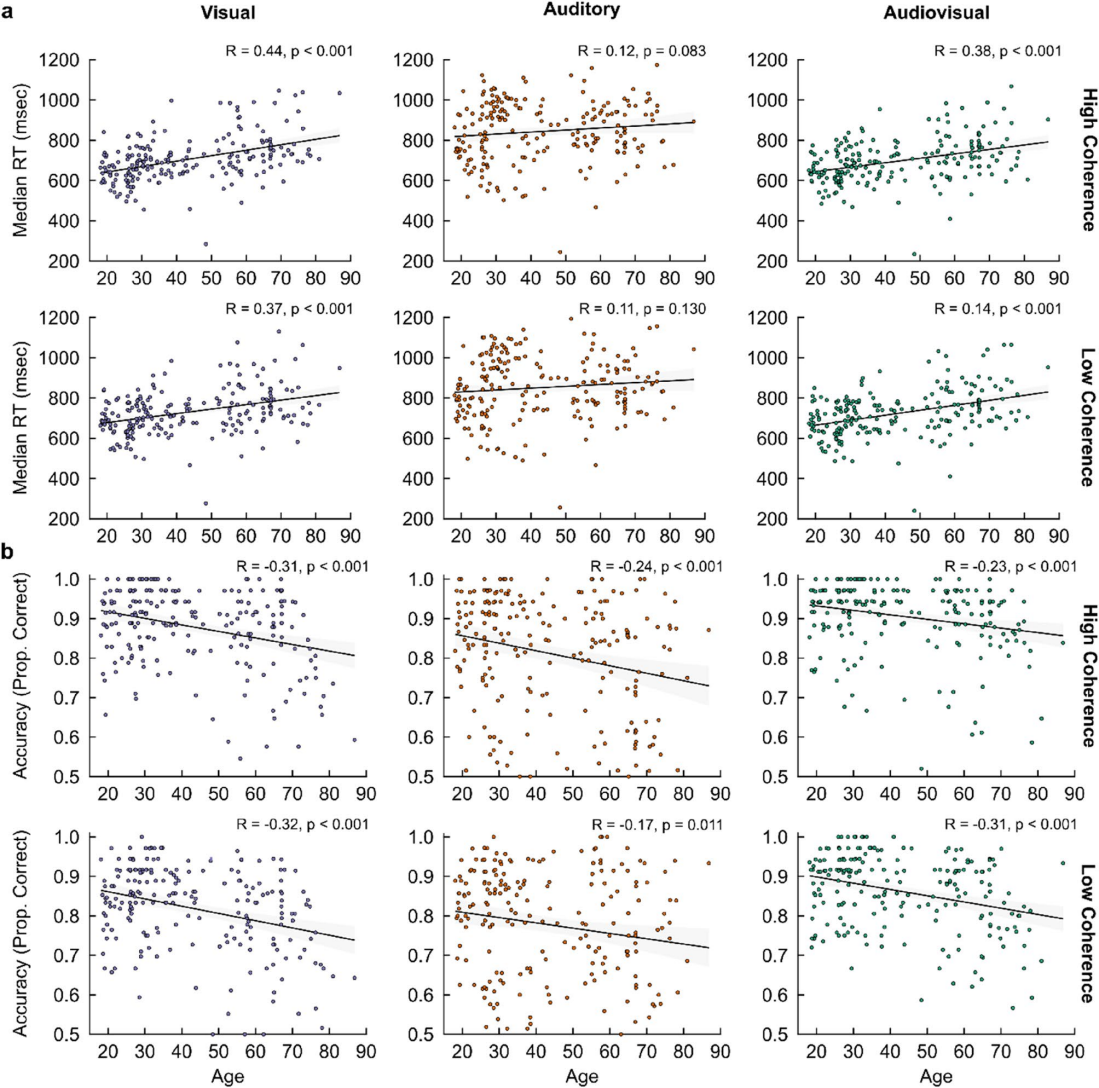
Behavioural performance—correlations with chronological age. Pearson correlations of age with (**a**) median RTs and (**b**) choice accuracy (proportion of correct responses) across the two levels of stimulus coherence (high/low coherence) as a function of visual (*left*), auditory (*middle*), and audiovisual (*right*) trials. Pearson Correlation Coefficients (R) and p-values are shown for each correlation. Shaded regions indicate 95% Confidence Intervals (CIs).

**Figure 3:**
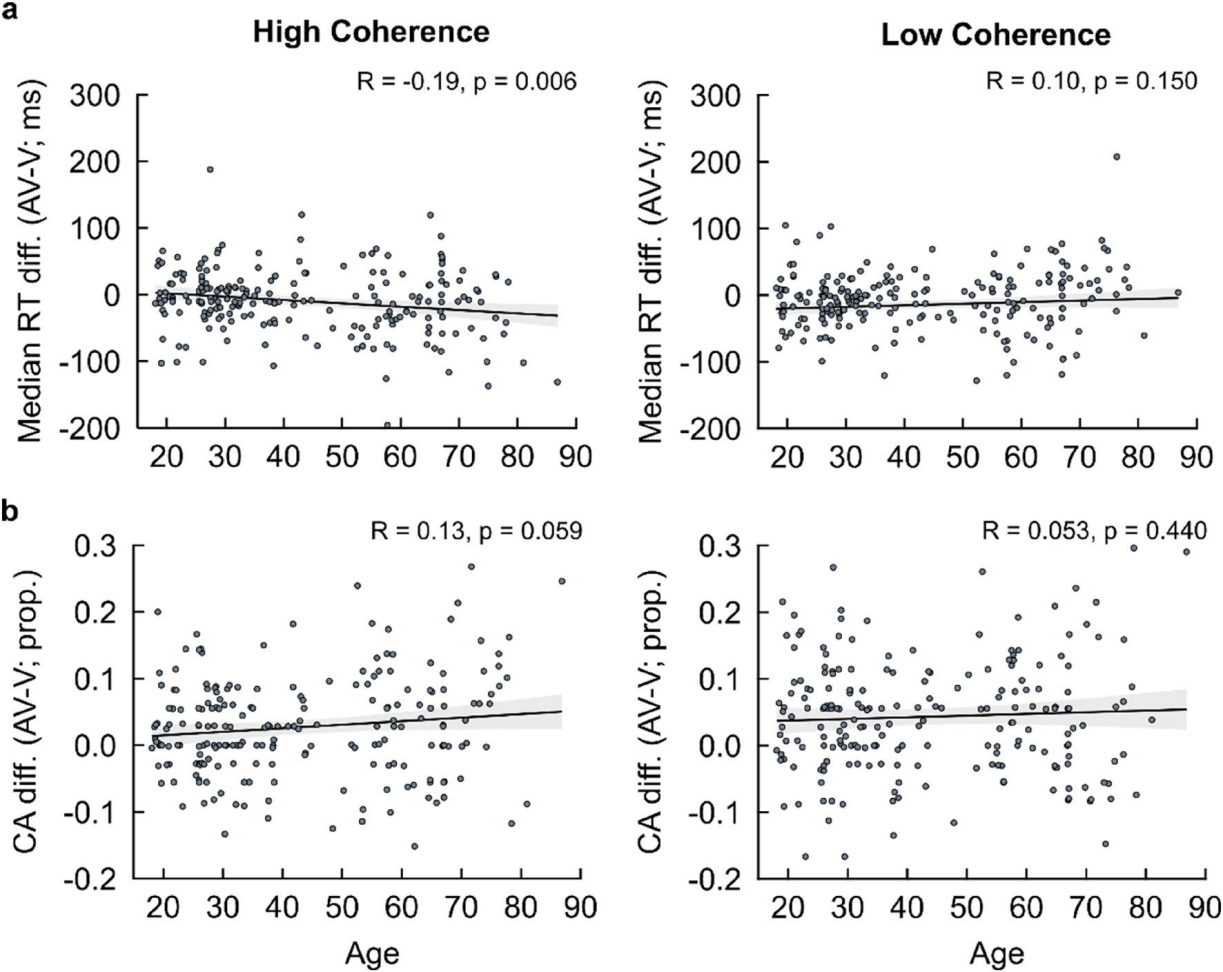
AV versus V behavioural performance differences—correlations with chronological age. Individual Pearson’s correlations of age with (**a**) median RT differences and (**b**) choice accuracy (proportion of correct responses) differences between AV and V conditions across the two levels of stimulus coherence (high/low coherence). Pearson Correlation Coefficients (R) and p-values are shown for each correlation. Shaded regions indicate 95% Confidence Intervals (CIs).

We found a significant main effect of age on RTs and choice accuracy, with RTs increasing (χ^2^ = 15.37, df = 1, p < 0.001, Fig. 2a) and choice accuracy decreasing (χ^2^ = 25.09, df = 1, p < 0.001, Fig. 2b) with age. Furthermore, a significant two-way interaction was demonstrated between age and sensory condition for RTs (χ^2^ = 129.13, df = 2, p < 0.001). Reduced comparisons between multisensory (i.e., AV) and unisensory (i.e., V and A) conditions revealed that, with ageing, a *larger* decrease in RTs was exhibited for AV compared to V conditions (χ^2^ = 6.27, df = 1, p = 0.012, Fig. 3a), whereas a *smaller* decrease in RTs was exhibited for AV compared to A conditions (χ^2^ = 69.16, df = 1, p < 0.001, Supplementary Fig. 6a) and for V compared to A conditions (χ^2^ = 106.59, df = 1, p < 0.001, Supplementary Fig. 6a). Regarding choice accuracy, no significant two-way interaction was demonstrated between age and sensory condition (χ^2^ = 4.18, df = 2, p = 0.124). This was reaffirmed with reduced comparisons between AV versus V conditions (χ^2^ = 0.57, df = 1, p = 0.452, Fig. 3b), AV versus A conditions (χ^2^ = 2.74, df = 1, p = 0.097, Supplementary Fig. 6b), and V versus A conditions (χ^2^ = 3.36, df = 1, p = 0.067, Supplementary Fig. 6b). Whereas no significant three-way interactions between age, sensory condition, and stimulus coherence were demonstrated for RTs (χ^2^ = 4.20, df = 2, p = 0.376) or choice accuracy (χ^2^ = 1.25, df = 2, p = 0.535), a significant reduced three-way interaction between AV versus V conditions alone (i.e., omitting A trials) was found for RTs (χ^2^ = 4.54, df = 1, p = 0.033), but not between AV versus A conditions (i.e., omitting V trials; χ^2^ = 0.39, df = 1, p = 0.532) or V versus A conditions (i.e., omitting AV trials; χ^2^ = 0.48, df = 1, p = 0.487).

Overall, we observed general age-related declines in decision speed (i.e., increased RTs) and accuracy (i.e., decreased proportions of correct responses). In addition, we saw age-related differences in RTs between multisensory (i.e., AV) versus unisensory (i.e., V and A) conditions. Specifically, older adults tended to display a multisensory benefit in RT differences between AV versus V conditions (i.e., larger AV–V RT differences), not seen otherwise between AV versus A conditions and irrespective of task difficulty. Coupled with this were no significant age-related impacts on choice accuracy between multisensory versus unisensory conditions. These findings suggest older adults display preserved, and somewhat enhanced, multisensory RT benefits (i.e., larger AV–V RT difference), particularly for complementary A evidence of decreased task difficulty (i.e., high stimulus coherence), alongside preservations in decisional accuracy.

### Hierarchical drift diffusion modelling results

**Figure 4:**
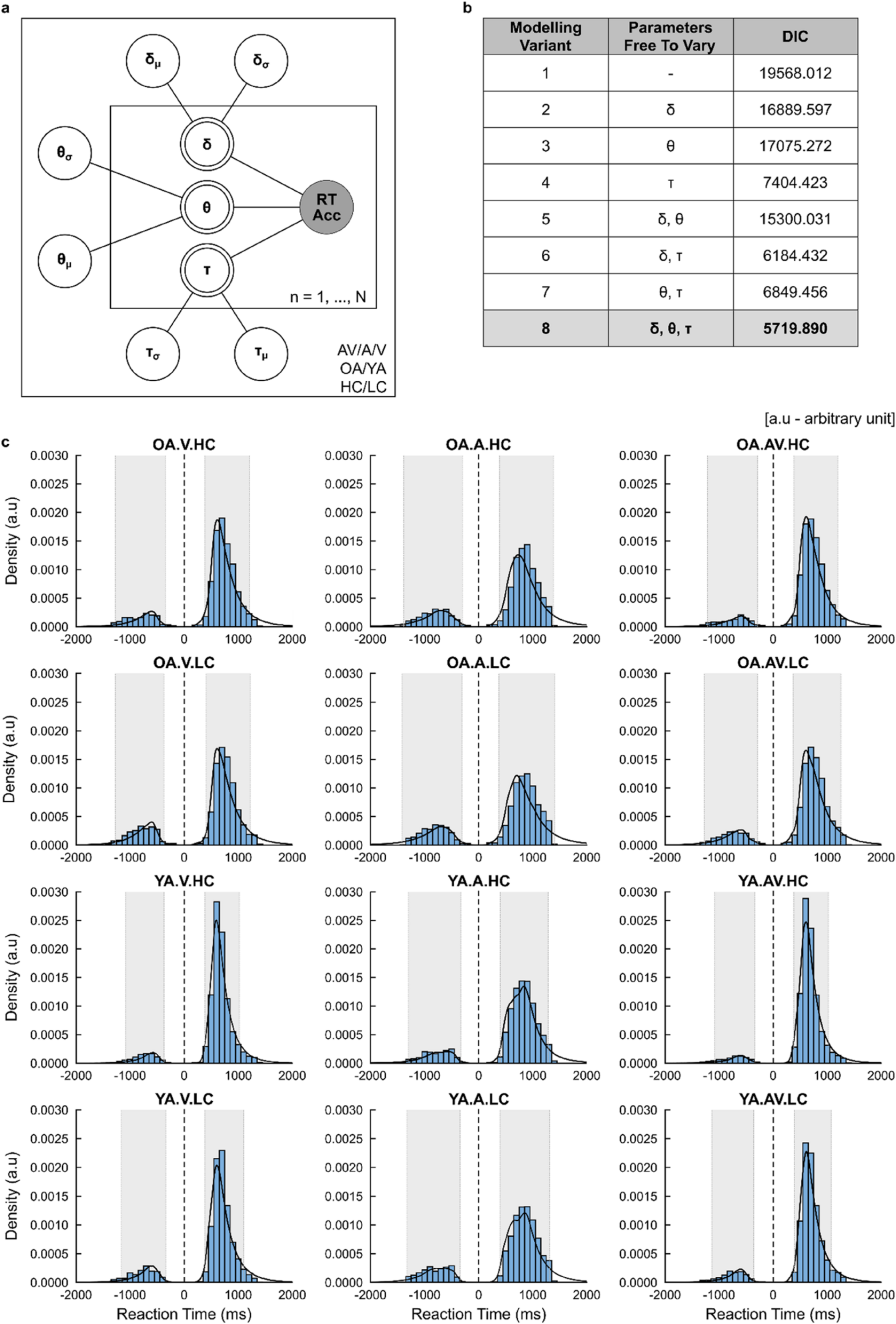
Hierarchical drift diffusion model—framework, fitting, and posterior predictive checks. (**a**) Graphical representation illustrating the Bayesian hierarchical framework for estimating behavioural HDDM parameters. Round nodes represent continuous random variables, with shaded nodes representing recorded variables, i.e., single-trial RTs and choice accuracy. Double-bordered nodes represent deterministic variables, defined in terms of other variables. Plates denote a hierarchical framework for modelling multiple random variables. The inner plate denotes participants (n = 1, …, N) and the outer plate denotes conditional dependencies for sensory conditions (Audiovisual; AV, Auditory; A, Visual; V), age group (Older Adults; OA, Younger Adults, YA), and stimulus coherence (High Coherence; HC, Low Coherence; LC). Combinations of drift rate (δ), decision boundary (θ), and non-decision time (τ) parameters are modelled as random variables with inferred means μ and variances σ and are constrained by inferred estimates over all conditional dependencies. (**b**) Deviance Information Criterion (DIC) scores and fixed parameters (i.e., free to vary across all conditional dependences) for all modelling variants tested. Shaded row denotes the best fitting Bayesian hierarchical modelling framework (i.e., lowest DIC score) towards behavioural data. (**c**) Posterior predictive checks of the best fitting HDDM for the behavioural dataset. Modelling fit to the behavioural dataset was assessed using combined histogram and density plots for RT distribution splits across all conditional dependences and accuracy of choice responses (positive and negative RT histogram/density plot distributions for correct and incorrect choice responses respectively). Histograms (in blue) and density plots (in black) denote observed and predicted RTs respectively. Dashed grey lines on the x-axis denote the credible intervals representing the Highest (posterior) Density Region (HDR) at a 90% confidence level for correct and incorrect choice responses.

Participants’ RTs and binary responses were then fit with HDDMs^39^ to return parameter estimates of the rate of evidence accumulation (i.e., drift rate, δ), the distance between correct and incorrect decision thresholds quantifying the amount of evidence required to facilitate one particular choice alternative (i.e., decision boundary, θ), and the duration of non-decisional processes (i.e., non-decision time, τ, Fig. 4a). Posterior predictive checks, simulating a behavioural dataset with the best fitting model (i.e., lowest Deviance Information Criterion: DIC, Fig. 4b) demonstrated a good fit with the observed empirical dataset (Fig. 4c, Supplementary Fig. 1).

We then sought to capture the key affected computations underlying significant age-related behavioural findings. Given that we observed participants tended to perform faster in V compared to A conditions (see Fig. 2a, Supplementary Fig. 5a and b), and coupled with our key behavioural findings (see “Behavioural results” section), we sought in particular how general age-related increases in decision speed (i.e., increased RTs) and decreases in choice accuracy can be reconciled against age-related linkages with (1) larger AV–V RT differences and (2) preservations in decisional accuracy. To achieve this, we performed correlations (using Pearson correlation coefficients) between each participant’s chronological (i.e., continuous) age with their respective HDDM posterior parameter estimations for drift rate (δ), decision boundary (θ), and non-decision time (τ), in order to assess the linear strength and direction of age-related impacts on HDDM parameters (see “Materials and methodology” section for further detailed information).

**Figure 5:**
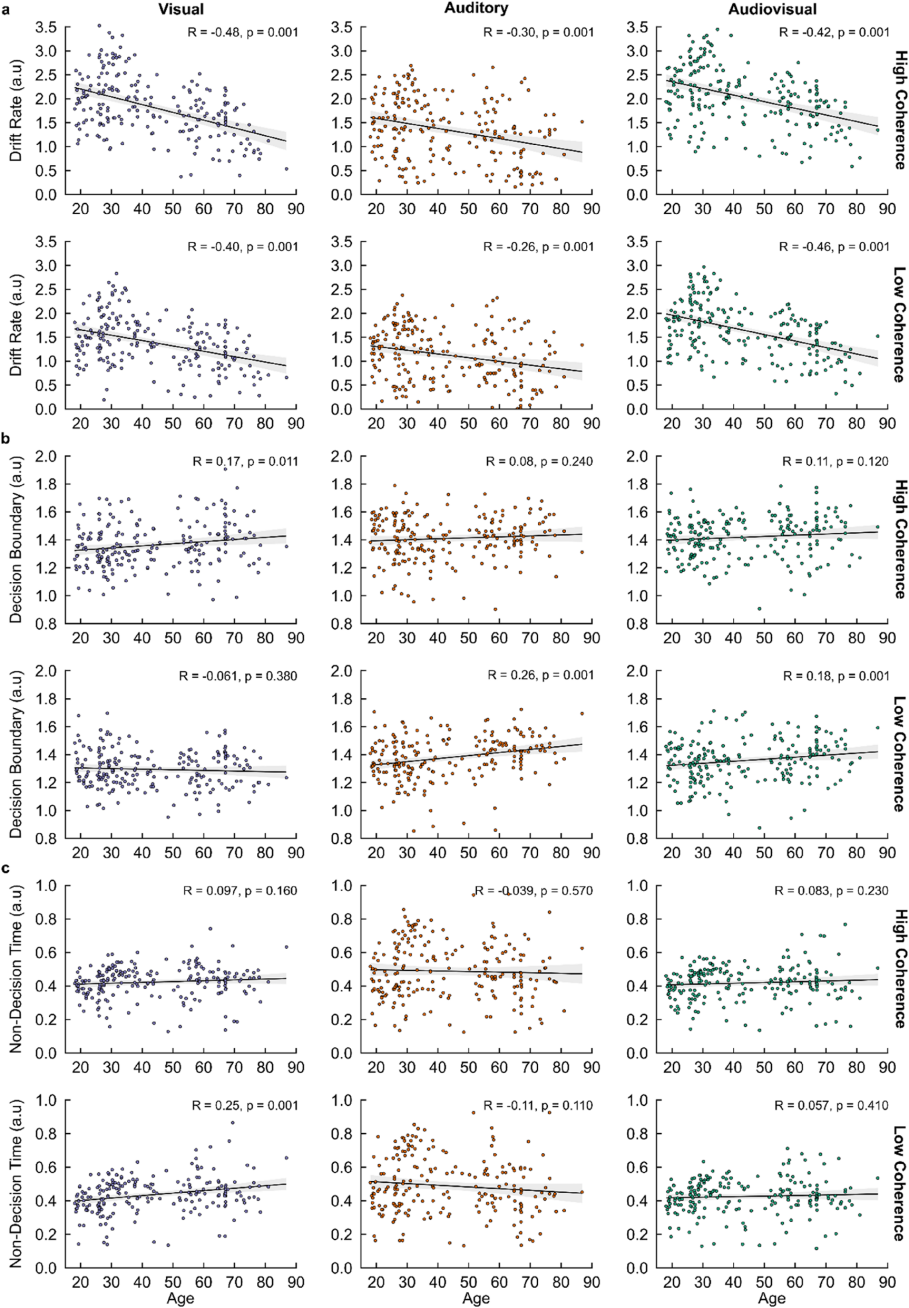
HDDM posterior parameter estimations—correlations with ageing. Individual Pearson correlations of age with (**a**) drift rate (δ), (**b**) decision boundary (θ), and (**c**) non-decision time (τ) parameters across all conditional dependencies (sensory condition: Visual, V; Auditory, A; Audiovisual, AV; stimulus coherence: High Coherence, HC; Low Coherence, LC; age range: Older Adults, OA; Younger Adults, YA). Pearson Correlation Coefficients (R) and p-values are shown for each correlation. Shaded regions indicate 95% Confidence Intervals (CIs).

First, our HDDM findings demonstrated a significant negative correlation of drift rate with age across all sensory conditions and stimulus coherence levels (i.e., p = 0.001, Fig. 5a) implying that sensory evidence accumulation slows with age. In addition, significant positive correlations of decision boundary with age for AV and A conditions within LC trials (Auditory/Low Coherence: R = 0.26, p = 0.001; Audiovisual/Low Coherence: R = 0.18, p = 0.001) and for V conditions within HC trials (Visual/High Coherence: R = 0.17, p = 0.011) were observed, alongside a significant positive correlation of non-decision time for V conditions within LC trials alone (Visual/Low Coherence: R = 0.25, p = 0.001, Fig. 5c). These findings suggest modality-specific increases in response caution (when processing AV and A stimuli) and sensory encoding (when processing V stimuli) are impacted due to increased task difficulty, coupled with an age-related increase in response caution when categorising more coherent visual representations, incurring costs to the time taken to facilitate reliable choice responses.

**Figure 6:**
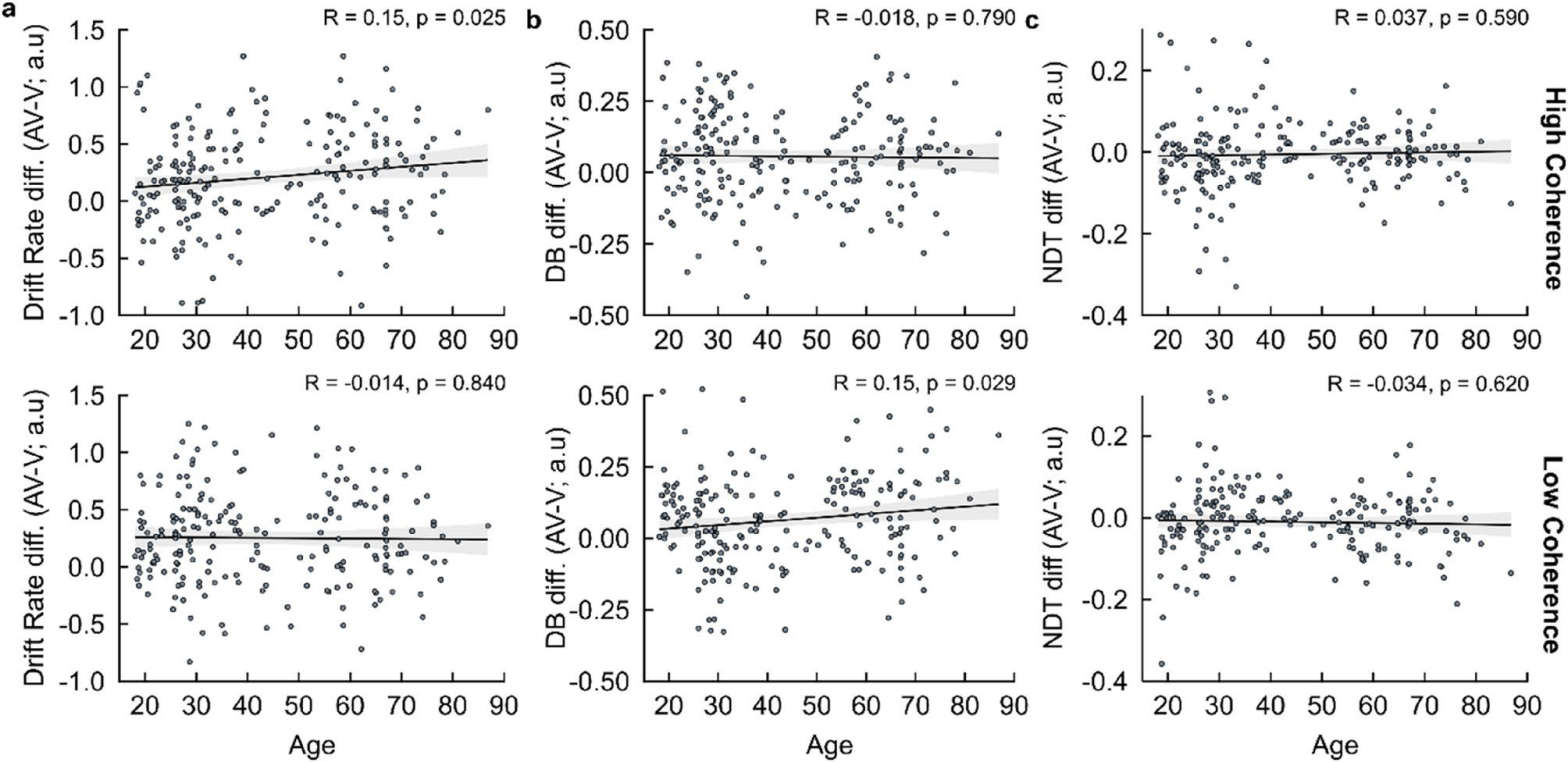
AV versus V trial type HDDM posterior parameter estimations—performance differences. Individual Pearson’s correlations of chronological age with (**a**) drift rate (δ), (**b**) decision boundary (DB, θ), and (**c**) non-decision time (NDT, τ) parameter estimate differences between AV and V trial types across levels stimulus coherence (high/low coherence). Pearson Correlation Coefficients (R) and p-values are shown for each correlation. Shaded regions indicate 95% Confidence Intervals (CIs).

Importantly, we then quantified multisensory benefits in the HDDM parameters and correlated them with chronological age to probe the effect of age on multisensory decision formation. We found a significant positive correlation with AV–V drift rate differences within HC trials (Audiovisual–Visual/High Coherence: R = 0.15, p = 0.025; Audiovisual–Visual/Low Coherence: R = − 0.014, p = 0.840, Fig. 6a). This implies that older adults display enhanced evidence accumulation with additional A evidence for trials of decreased task difficulty (i.e., HC trials), which is consistent with the significantly greater RT difference exhibited in our behavioural results. Coupled with this was a significant positive correlation for AV–V decision boundary differences for LC trials (Audiovisual–Visual/High Coherence: R = − 0.018, p = 0.790; Audiovisual–Visual/Low Coherence: R = 0.15, p = 0.029, Fig. 6b). These results suggest an age-related increase in response caution when complementary A evidence is consolidated with increased task difficulty (i.e., LC trials). Given we observed in our behavioural results that ageing was associated with a greater multisensory AV-V benefit towards RTs, coupled with a significant reduced three-way interaction suggesting such multisensory benefits are impacted within LC trial types (see “Behavioural results” section), older adults are more likely to display increased caution in choice responses when complementary auditory evidence is more difficult to categorise, thus preserving decision accuracy.

### Optimal drift rate coefficient differences: correlations with chronological age

**Figure 7:**
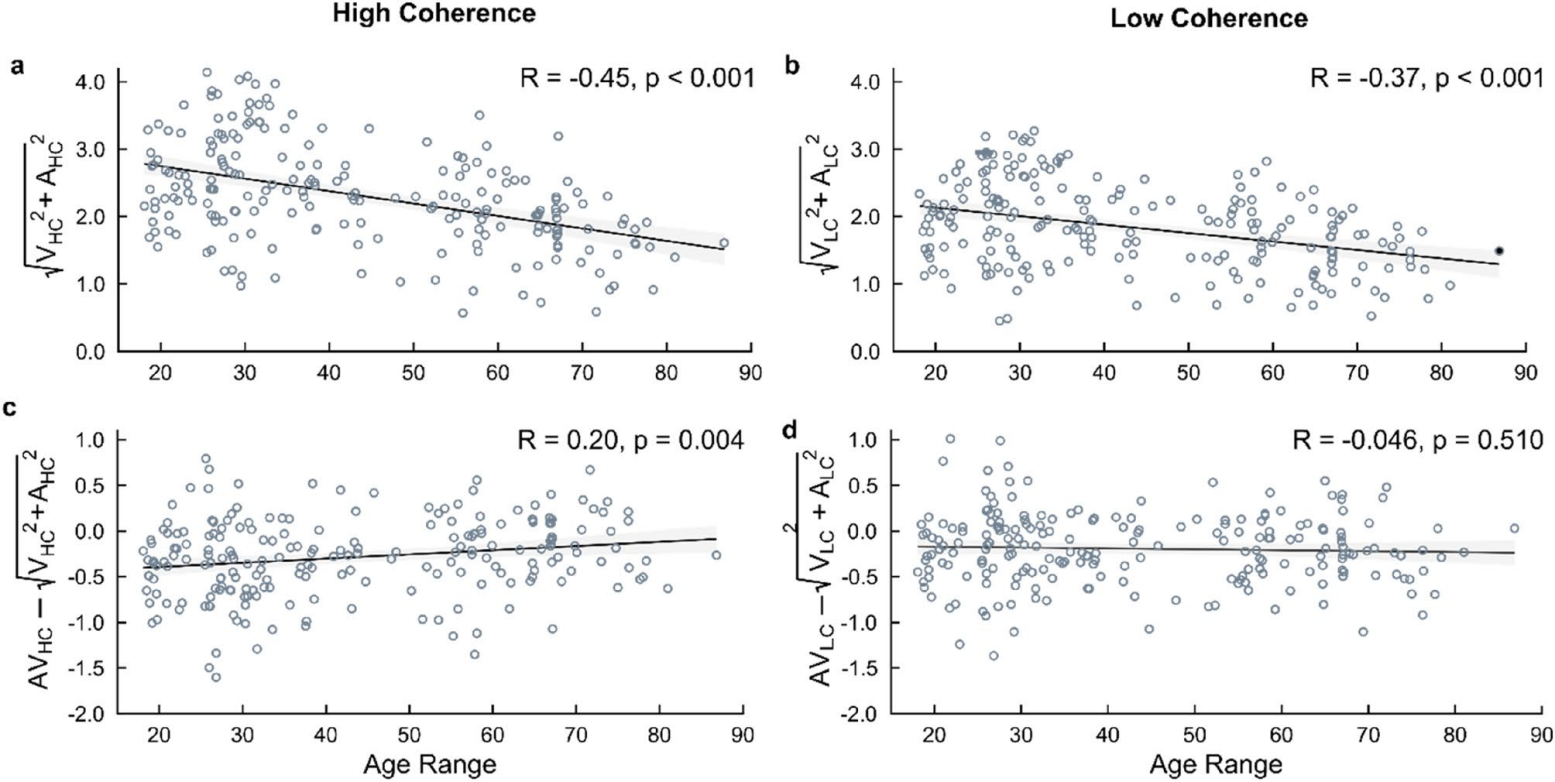
HDDM drift rate posterior parameter differences—correlations with chronological age. Individual participant correlations of chronological age with (**a**,**b**) predicted multisensory cue combinations (optimal combination of unisensory cues), and (**c**,**d**) observed multisensory minus predicted cue combinations, of drift rate (θ), as derived by Drugowitsch et al.^20^, across two levels of stimulus coherence (*left*: High Coherence; *right*: Low Coherence). Pearson Correlation Coefficients (R) and p-values are shown for each correlation. Shaded regions indicate 95% Confidence Intervals (CIs).

To further examine if the age-related increases in multisensory benefit between AV and V conditions generalise across sensory conditions, we compared the participants’ multisensory drift rates with the optimal combination of their two unisensory drift rates (see “Materials and methodology” section). The optimal combinations of unisensory drift rates demonstrated significant negative correlations with chronological age (HC trial types; R = − 0.45, p < 0.001; LC trial types; R = − 0.37, p < 0.001, Fig. 7a and b), reaffirming HDDM findings of older adults exhibiting lower drift rates across unisensory trials relative to younger adults (see *Hierarchical Drift Diffusion Modelling Results*). Differences between observed multisensory (i.e., AV) and optimally combined unisensory drift rates, however, yielded a significant positive correlation with age for trials of lower difficulty (HC trial types; R = 0.20, p = 0.004, Fig. 7c), but no significant correlation for higher difficulty trials (LC trial types: R = − 0.046, p = 0.510, Fig. 7d). Therefore, in easier trials older adults exhibit an increased likelihood of a multisensory benefit.

### Effect of chronological age on the principle of inverse effectiveness

**Figure 8:**
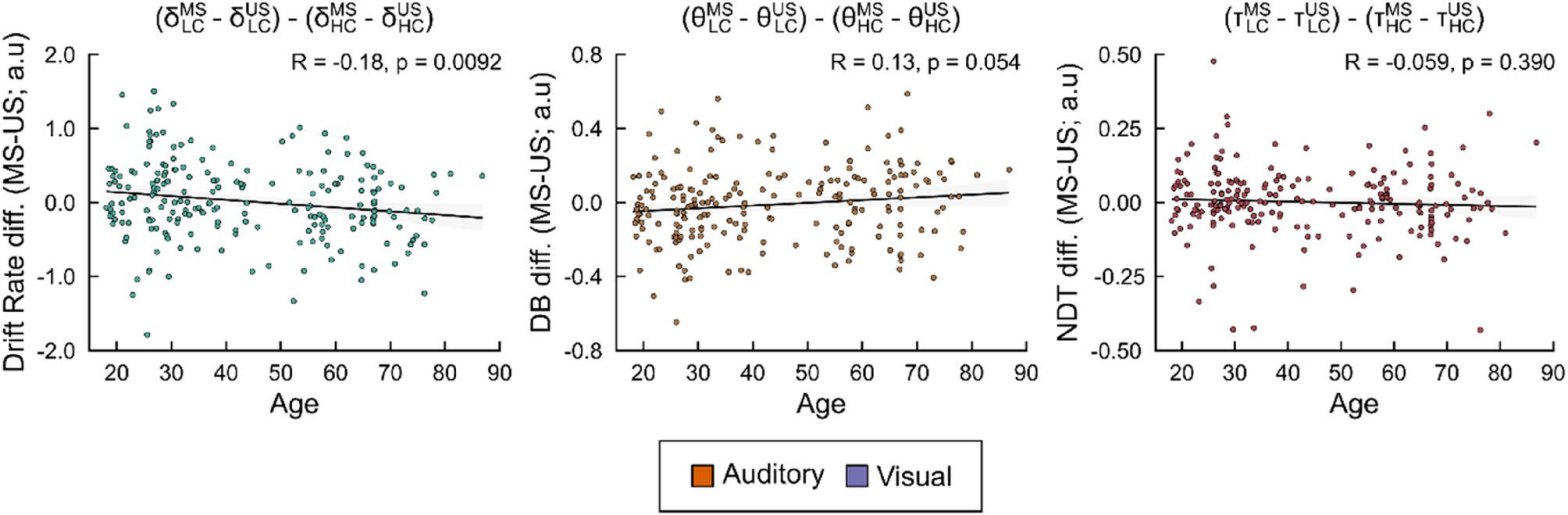
HDDM stimulus coherence-based posterior parameter differences—correlations with chronological age. Individual participant correlations of age with drift rate (*left*; δ), decision boundary (*middle*; DB, θ), and non-decision time (*right*; NDT, τ) stimulus coherence-based posterior estimation differences (i.e., LC–HC) between multisensory (MS) versus unisensory (US) trial types of optimal modelling performance (i.e., highest drift rate, lowest decision boundary, and lowest non-decision time respectively). Pearson Correlation Coefficients (R) and p-values are shown for each correlation. Shaded regions indicate 95% Confidence Intervals (CIs).

We then tested if age-related multisensory benefits could be attributed to the Principle of Inverse Effectiveness (tPoIE) in multisensory processing which suggests that benefits are stronger when unisensory evidence (stimulus coherence here) is weaker. When associating the measure of inverse effectiveness (MIE—see “Materials and methodology” section for details) with the participants’ age, a significant negative correlation was found for drift rates (R = − 0.18, p = 0.0092, Fig. 8*left*), but not for decision boundary (R = 0.13, p = 0.054, Fig. 8*middle*) or non-decision time (R = − 0.059, p = 0.390, Fig. 8*right*). This suggests that the effect of tPoIE in multisensory evidence accumulation decreases with chronological age.

## Discussion

In this study, we coupled single-trial behavioural metrics of multisensory decision-making, recorded from an online audiovisual object categorisation paradigm^2^, with Hierarchical Drift Diffusion Modelling (HDDM^39^) to assess age-related impacts on the latent cognitive processes underlying multisensory decision-making. This methodology provided a principled and coherent account for characterising age-related modulations within the formation of perceptual decisions. Furthermore, it offered a mechanistic insight into the utilisation of multisensory information for perceptual decision-making and its changes with age. Consequently, we could address twofold aims: (a) investigate age-related differences in recorded behavioural indices between trial types, and (b) dissect the constituent processes that captured such age-related differences, allowing us to probe the internal components that are likely to remain either preserved or degraded in older adults. In particular, we demonstrated that (a) whereas overall, older adults were slower (i.e., ↑ RTs) and less accurate (i.e., ↓ choice accuracy) across all sensory conditions (Fig. 2), they exhibited greater decreases in RTs, coupled with no significant effects of choice accuracy, between AV versus V conditions (i.e., ↑ AV-V RT difference, Fig. 3). Here, we capture multisensory benefits towards decisional speed alongside a preservation of decisional accuracy. HDDM demonstrated parsimonious fittings for characterising such behavioural discrepancies as a function of age. Notably we found (b) slower rates of sensory evidence accumulation (i.e., ↓ drift rates) for older adults across all sensory conditions, coupled with higher rates of sensory evidence accumulation (i.e., ↑ drift rates) for older adults between AV versus V conditions of decreased task difficulty, coupled with increased response caution (i.e., ↑ decision boundaries) between AV versus V conditions of increased trial difficulty.

The observation of older adults exhibiting lower decisional speed (i.e., higher RTs) and accuracy (i.e., lower proportion of correct responses) across all sensory conditions, irrespective of stimulus coherence, supports previously published research showing that older adults typically exhibit larger RTs in speeded paradigms relative to younger adults^13–15^. Coupled with declines in speeded choice accuracy^43^, this further suggests that older adults have slower decisional speed in consolidating decisional evidence for choice formation, as evidenced by slower uptakes in sensory evidence accumulation observed in our HDDM. Previous ageing DDM analyses have reported discrepancies in drift rate findings^13–15^. For instance, older adults have been demonstrated to exhibit lower drift rates in paradigms assessing letter discrimination^38^, similar drift rates in signal detection paradigms^44,45^, and even higher drift rates in motion discrimination (albeit with a Linear Ballistic Accumulator model) respectively^32^. Given our contrasting observations of consistent age-related decreases in drift rate for simple face-versus-car object recognition, we attribute the variability in drift rate findings across experimental paradigms and stimuli to behavioural processes that go beyond simple categorisations, impacting how the presented sensory evidence must be encoded and accumulated to facilitate choice responses. In comparison with the findings of Jones et al., who reported an increased likelihood of older adults encoding noisier sensory representations in audiovisual spatial localisation (a more complex categorisation task)^19^, we recommend further study comparing the within-trial dynamics of multisensory drift rate (and thus evidence accumulation) between simple and more complex object categorisation tasks.

In addition, age-related increases in response policy caution (i.e., decision boundary) for A and AV conditions of increased trial difficulty (i.e., LC trials) were observed. However, we did not display consistent findings suggesting a slowing of sensory encoding nor motoric response execution (i.e., non-decision time). Elevated decision boundaries and sensory processing have previously been implied to be consistent processes underlying age-related decreases in perceptual decision-making speed, owing to a developmental increase in cautiousness^13,30,31,33,36,38,46–49^. In consideration of such trends, it has been thought they exhibit increased differences in boundary settings due to sensory encoding limitations (thus impacting non-decisional processing times), or strategies voluntarily applied, to preserve multisensory integrative benefits towards choice behaviour^13^. Our HDDM findings support the latter, further highlighting the effect of task difficulty increasing sensory evidence uptake to facilitate perceptual decision formation, yet not so much in circumstances where simple categorisations suffice across sensory modalities, or when one modality displays lessened reliability in sensory representation, as evidenced by the observation of age-related increases in response policy caution for V trials of decreased task difficulty (i.e., HC trials).

Our core observations highlighted that older adults exhibited significantly greater AV–V RT differences, alongside a significant age-related increase in AV–V RT differences when complementary unisensory information was of increased evidence salience. This was further coupled with no significant AV–V choice accuracy differences (see Fig. 3c and d), further highlighting a preservation in decisional accuracy irrespective of task difficulty. HDDM hypotheses testing of AV versus V differences reaffirmed that older adults were more likely to benefit from an increased rate of sensory information uptake (i.e., increased AV–V drift rate differences) when consolidating complementary auditory information in the accumulation of visual evidence, irrespective of task difficulty when assessing categorical age (see Supplementary Materials S6 and S7). In addition, they demonstrated an increase in response caution (i.e., increased AV–V decision boundary differences) when task difficulty increased, and complementary information was therefore more difficult to reconcile. In line with the increase in decision thresholds, observed in Jones et al.^19^, predicting increased RTs for increasingly disparate AV spatial localisations, we offer further modelling insights reconciling why older adults may display preserved (and to an extent enhanced) multisensory integrative benefits in light of inherent perceptual declines towards decision formation, capturing an interplay between the uptake of sensory evidence accumulation and response caution in decision policy that varies in accordance with task difficulty (i.e., stimulus coherence). In the context of the experimental paradigm utilised^2^, it appears visual representations of faces and cars are amplified by the complementary auditory evidence driving downstream audiovisual RT improvements for older adults, as well as post-sensory decision dynamics arising to preserve choice accuracy when complementary auditory evidence becomes increasingly difficult to reconcile.

Chiefly, our comparisons of the multisensory (i.e., AV) drift rates with the optimal consolidation of the complementary unisensory (i.e., V + A) drift rates across stimulus coherence (i.e., HC/LC) trials, adopting the equation derived by Drugowitsch et al.^20^ (Fig. 7), reflect that older adults are more likely to benefit from an optimal cue-combined sensory evidence accumulation of decreased task difficulty (i.e., HC trials; Fig. 7a and b). These increased drift rate coefficient differences, across the adult lifespan, are likely to be reduced when cue-combined sensory evidence accumulation is of increased task difficulty, but preserved from the impact of task difficulty (i.e., LC trials; Fig. 7c and d). A hypothesis in the field; the *general cognitive slowing* hypothesis^10,12^, has previously posited that age-related multisensory integrative enhancements are an artefact of increased cognitive demand in processing independent unisensory signals which provide redundant information about the same multimodal object (i.e., same stimulus properties presented to different sensory modalities^49–52^. However, it has been previously argued that this hypothesis cannot fully justify why multisensory integrative benefits remain intact, and to an extent enhanced, across the adult lifespan^10,53,54^. Rather, it has been found that signal intensities (i.e., stimulus coherence) impact this notion, and most prominently impact unisensory “baseline” processing levels^55–57^, as well as top-down attentional control^58,59^ despite the increased likelihood of reduced cognitive demand. In the HDDM space, we suggest that such slowing effects in sensory processing of multiple stimuli would primarily influence non-decision times (i.e., stimulus encoding delays). Given we did not observe any prominent age-related impacts on non-decision time differences between AV versus V conditions, and the correlation of cue-combined unisensory drift rates for LC trials did not exhibit a significant direction in trend, we suggest that a general cognitive slowing remains for consolidating independent unisensory stimuli, but does not result in artificially enhanced multisensory integrative benefits observed in previous research^7–9^. Furthermore, signal intensities do not solely impact earlier sensory encoding processes (i.e., non-decision time), nor degrade later post-sensory decision dynamics (i.e., drift rate and decision boundary), but are inherent in the cue-combination of unisensory signals when such complementary unimodal information becomes increasingly difficult to consolidate, or when modalities exhibit discrepancies in reliability of sensory representation^60,61^.

Further insights empirically validating this notion concern the governing *principle of inverse effectiveness* (tPoIE) for multisensory integration^62–64^. tPoIE outlines that the magnitude of multisensory enhancements increases when the effectiveness of processing unisensory stimuli decreases. Therefore, less salient unisensory stimuli are more likely to be integrated, and more salient unisensory stimuli less likely to be integrated, in order to benefit perceptual decision formation. Given research demonstrating age-related functional deficits in sensory systems (e.g., visual acuity^65,66^; auditory pure-tone hearing thresholds^67,68^), multisensory enhancements towards perceptual decision formation are likely to remain preserved, or subsequently increased, in older adults due to reduced acuity in individual senses^13^. This would result in increased multisensory benefits towards choice selection as the stimulus coherence of unisensory stimuli remains naturally degraded^7,8^. Our HDDM findings uncovered an increased likelihood of tPoIE that did not impact non-decision time (see Fig. 8c *right*), arguing against its impact on sensory encoding and/or motor production latency, nor decision boundary (see Fig. 8*middle*), impacting the distance between choice boundaries, but rather drift rate (see Fig. 8*left*). Interestingly, our measure of tPoIE correlated positively with age suggesting a decrease (rather than increase, as is commonly hypothesised^69,70^) in the effect of tPoIE on evidence accumulation with age. Given that multisensory benefits remained preserved when information became increasingly difficult to reconcile through AV preservations in drift rate and increases in decision boundary, we suggest that tPoIE decreases in likelihood to benefit multisensory integration across the adult lifespan in decisional speed, and is compounded by a compensatory mechanism in response caution that necessitates the need for additional complementary unisensory information to be accumulated to preserve choice accuracy.

In conclusion, we demonstrated novel insights into the key computations determining preserved multisensory benefits within perceptual decision formation in older adults despite inherent declines in perceptual decision-making. Notably, we characterised that despite an age-related slowing of sensory information processing for both multisensory and unisensory information, older adults still exhibit multisensory integrative benefits. Namely, they benefit from an increased uptake in sensory evidence accumulation to benefit decisional speed. Furthermore, they exhibit increased decision policy caution when faced with a decreased salience in stimulus properties, and therefore increased task difficulty, to preserve such benefits when decisional speed is compromised. Given older adults have been found to exhibit, for example, increased predispositions to fall^71^, as well as increased difficulty in audiovisual speech perception due to demands in processing dynamic cues^70,72^, our results demonstrate the importance of modelling age-related impact on multisensory decision-making behaviour. As such, we recommend further exploration to parse apart ageing effects on the precision of (multi)sensory stimulus representations and how they are modulated by difficulty in evidence consolidation, perhaps by (mis)matching difficulty levels across sensory modalities^2^. We advocate for this at both a computational and cortical level to further incorporate age-related changes to the brain that may alter regions underlying multisensory integrative and decision-related processes^12^.

## Materials and methodology

### Participants

An a priori power analysis was conducted using G*Power (version 3.1.9.7^73,74^) to estimate the minimum sample size required to test the study hypotheses. Our analysis indicated that for three predictors, a minimum sample size of 176 participants was required to achieve 95% power (i.e., β = 0.95) for detecting a moderate effect size (i.e., f^2^ = 0.10; see^75^) at a significance criterion of α = 0.05. Therefore, a sample of 212 participants (male = 105, female = 107; M ± SD = 43.52 ± 18.46, age range = 18.08–86.83 years) was selected from an initial pool of 357 participants after completing the full experiment (see “Statistical analysis of behavioural data” section) on the Gorilla Experiment Builder research platform (http://www.gorilla.sc^41,42^), receiving a £10 (UK Sterling) Amazon Voucher as payment. All selected participants gave their informed consent prior to participation, self-reported normal hearing and normal or corrected-to-normal vision, and no history of neurological deficits. This study was approved by the Research Ethics Committees of the College of Business, Law, and Social Sciences at Nottingham Trent University (BLSS REC 2021/45) and the Faculty of Biological Sciences at the University of Leeds (BIOSCI 19-021). It was conducted in accordance with the Declaration of Helsinki^76^.

### Stimuli

We used a set of 36 grayscale images as the visual stimuli—18 of faces and 18 of cars (image size: 512 × 512 pixels; bit depth: 8 bits/pixel)—sourced and adapted from previous experiments^2,17,40,77–80^. Previous experiments sourced the original face images from the Face Database of the Max Plank Institute of Biological Cybernetics^81^ and the original car images from the Internet^2^. For each retrieved image, the background was removed, and the image transferred onto a uniform grey background. All images were equated for spatial contrast, frequency, luminance, and total number of frontal and side views (a maximum of ± 45°), and all had identical magnitude spectra (i.e., average magnitude spectrum of all images in the database), with their corresponding phase spectra manipulated using the weighted mean phase technique^82,83^. This technique alters image phase coherence and characterises phase coherence percentage, therefore altering the amount of visual sensory evidence in the stimuli available. To manipulate paradigm difficulty, we used two levels of visual sensory evidence (32.5% and 37.5% phase coherence). These levels are based on previous studies and are known to result in performance spanning the psychophysical threshold^2,17,40,77–80^. All images were displayed on a white background (RGB: [255 255 255]) for a duration of 300 ms and were developed using the PsychoPy software (version 1.82.01^84^).

In addition, we used 36 sounds as the auditory stimuli—18 of human speech and 18 of traffic sounds (adapted from^2^). They were presented alone or in addition to visual stimuli on one third of trials. All sounds were sourced from Franzen et al.^2^. No copyright restrictions were in place and sound file modifications were permitted. All sound files were sampled at a rate of 22.05 kHz and stored as .wav files. A 10 ms raised-cosine on/off ramp filter was added using MATLAB (version 2015b; The Mathworks, 2015, Natwick, Massachusetts) to reduce the effects of sudden sound onsets, with all sounds normalised by their standard deviation (SD). Normalised sound amplitudes were reduced by 80%, therefore lowering their intensity. Sounds were then embedded in Gaussian white noise, with the relative amplitude of sounds and noise manipulated to create two different levels of relative signal-to-noise (SNR) ratios, and therefore paradigm difficulty. This corresponded to two levels of sound phase coherence, therefore altering the amount of auditory sensory evidence in the stimuli available (0% and 25% SNR ratios). The resulting noisy speech and traffic sounds were presented binaurally for a duration of 300 ms.

### Experimental paradigm and procedure

We employed a modified variant of the audiovisual face-versus-car object categorisation paradigm^2,40^ (see Fig. 1a). This is a simple categorisation task that requires participants to classify whether a face or a car is embedded in a presented stimulus. Presented stimuli consisted of (1) face and car images (V trials), (2) human speech and traffic-related sounds (e.g., car horns or slammed doors; A trials), or (3) simultaneously presented images and sounds of faces/human speech and cars/traffic-related sounds (AV trials). AV stimuli were always semantically compatible, and image-sound mappings were never mismatching. All stimuli were presented for a duration of 300 ms in a pseudorandomised sequence. Two levels of phase coherence (High Coherence: HC/Low Coherence: LC levels respectively, Fig. 1b) were used to manipulate the difficulty of object categorisations. Participants were asked to indicate their decision via button press on a standard keyboard as quickly and as accurately as possible. The response deadline was set at 3000 ms. Reaction times (RTs; ms) and binary responses (a metric of choice accuracy) were recorded as single-trial dependent variable measurements quantifying behavioural performance (and perceptual decision formation).

The experimental paradigm was prepared using the Gorilla Experiment Builder research platform (http://www.gorrila.sc^41,42^). It was available to complete through an online URL, which was advertised using social media. The experiment could only be completed on a standard desktop or portable laptop computer. Prior to participation, participants were presented with pages detailing ethics, study information, and instructions for preparation prior to participation. Specifically, participants were instructed to position themselves in a quiet environment, with minimal distractions, and to use headphones or a sound system set at an appropriate volume to adequately hear sounds. Then, participants read instructions outlining the paradigm itself, in which they would be shown a quick and distorted (i.e., “noisy”) sequence of images only (V trials), sounds only (A trials), and images and sounds together of faces/human speech or cars/traffic-related noises (AV trials). They were asked to decide whether they identified a face or a car in the presented stimulus. They were instructed to position their left index and middle fingers of their right hand over the *j* and *k* standard keyboard buttons respectively, and to make their decision as quickly and as accurately as possible, pressing *j* for face stimuli and *k* for car stimuli. Participants were informed that audiovisual stimuli would always be matching (i.e., congruent; faces-human speech, cars-traffic sounds) and that images and sounds would never be mismatching (i.e., incongruent; faces-traffic sounds, cars-human speech). Participants were also instructed to refrain from categorising images and sounds individually in these trials, and to divide attention equally when basing their decision on visual and auditory information. Participants were asked that if they were unsure about their decision they were to guess to the best of their capabilities, since they had a maximum time limit of 3000 ms to make their response, with visual feedback presented for 1000 ms following each response.

Figure 1a illustrates the procedure on a single-trial basis. Each trial started with a black (RGB: [0 0 0]) fixation cross presented centrally on-screen for 1000 ms. Next, one of three stimuli (V, A, AV trials) was presented for a duration of 300 ms. Auditory stimuli were accompanied by an image of a speakerphone on-screen, which indicated the presented stimulus was a sound. Participants would then categorise, as quickly and as accurately as possible, the stimulus object as a face or a car, using the correctly assigned standard keyboard button (i.e., *j* and *k* keyboard button presses for face and car stimuli respectively). Feedback was presented centrally on-screen for 1000 ms for two possible outcomes: (1) a tick in green (RGB: [3 129 3]) for correct responses, or (2) a cross in red (RGB: [160 0 0]) for incorrect responses. In total, we presented 216 trials, which were presented in three blocks of 72 trials each and divided equally between the sensory conditions (i.e., 24 V trials, 24 A trials, and 24 AV trials per block), with a 60 s rest period between blocks. Furthermore, all trials were divided equally between the two stimulus object categories (i.e., 108 face trials and 108 car trials) and the two levels of stimulus coherence (i.e., 108 HC trials and 108 LC trials). The entire experiment lasted approximately 20–25 min.

### Statistical analysis of behavioural data

For each participant, RTs (calculated in ms) and choice accuracy (calculated as a binary variable of correct and incorrect responses) were collected as single-trial dependent variable measurements quantifying behavioural performance (and perceptual decision formation) for three categorical independent variables: (1) sensory condition (three levels: Visual, V trials; Auditory, A trials; Audiovisual, AV trials), (2) stimulus coherence (two levels: High Coherence, HC; Low Coherence, LC), and (3) chronological age, quantified as a continuous variable, whereby age in years and months (as of task completion) was computed as a decimal.

Our initial sample of 357 participants were screened to ensure they demonstrated a full, honest, commitment towards completing the full experiment to the best of their capabilities. Specifically, we excluded participants’ attempts to complete the experiment more than once, and excluded participants who did not meet a criterion of 50% correct responses across all sensory conditions, demonstrating behavioural performance above a baseline chance level (i.e., guesses), and ensuring participants did not procure timed-out responses (i.e., a maximum RT of 3000 ms) in the majority of, if not all, trials. This resulted in our sample of 212 selected participants who satisfied our exclusion criteria. For each participant, trials with RTs that exceeded median RT ± 2.5 Median Absolute Deviations (MADs), including trials where no response was made within the 3000 ms time-limit, were excluded from further analyses, with these RTs attributed to outliers corresponding to “fast guesses” or attentional lapses during testing^85^. This pre-processing criterion was selected as it has been demonstrated that MADs are a more robust measurement of central dispersion than standard deviation^86^. In total, 4027 trials were excluded from an initial 45,792 trials, leaving 41,765 trials for further analyses.

Our main statistical analysis quantified participants’ behavioural performance using Generalised Linear Mixed-Effects Models (GLMMs), which were applied using the *lme4* package^87^ in RStudio (R Core Team, 2022). GLMMs are considered preferable to use over conventional repeated-measures (M)ANOVA statistical analyses, due to their principled methodologies for modelling non-spherical error variance and heteroscedasticity^88,89^. In particular, random effects structures can be incorporated into the design of a GLMM to account for inter-individual and inter-predictor variability around population-level average effects, therefore increasing statistical power. In addition, GLMMs permit for the mixing of categorical and continuous variables in the statistical analysis of outcome variables, which themselves may be categorical or continuous, and can flexibly accommodate different types of outcome distributions through the application of a variety of link functions^90,91^. This permits us to therefore analyse age both as a split chronological (i.e., continuous) and categorical variable (see Supplementary Materials and Figures).

Our GLMMs included main effects and interactions of the three predictors: sensory condition, stimulus coherence, and chronological (i.e., continuous) age as predictor variables, along with by-participant random slopes and random intercepts. Random correlations were excluded for all GLMMs. This random effects structure was justified by our experimental design and adopted to ensure parsimonious fits of our GLMMs to the behavioural dataset. We specified *gamma* and *binomial logit* GLMMs for RTs and binary responses respectively. All categorical predictors were entered in mean-centred form using deviation coding. By using mean-centred contrast coding schemes, small imbalances in trial numbers between each predictor’s levels (and their interactions) can be accounted for. All GLMMs were fit using a *bobyqa* optimizer to ensure model convergence. Likelihood-ratio (χ^2^) model comparisons were used to quantify the predictive power and significance of all main effects and interactions in our GLMM analyses, and further reduced to quantify the predictive power and significance between two out of three levels of sensory trial type at a time. These likelihood-ratio (χ^2^) model comparisons compared full models (i.e., models including main effects, their two-way interactions, their three-way interactions, and random effects) to reduced models that excluded the main predictor, two-way interaction, or three-way interaction in question. In particular, we sought to investigate age-related differences in RTs and proportions correct between multisensory (i.e., AV trials) versus unisensory (i.e., V or A trials) conditions. Therefore, we primarily focused on main effects of our age predictor variable and their interactions with sensory condition and/or stimulus coherence.

### Hierarchical drift diffusion model—description

We fit participants’ RTs and binary responses with *Hierarchical Drift Diffusion Models* (HDDMs)^39^. Similar to traditional *Drift Diffusion Models* (DDMs)^24–29^, HDDMs shape perceptual decisions as a stochastic process of evidence accumulation indicative of one of two forced choice alternatives (e.g., correct/incorrect responses; left/right keyboard button presses), with accumulated evidence sequentially evaluated over time. For each decision process, the HDDM returns estimates of four parameters that prominently define the scope of the internal components capturing perceptual decision formation: (1) the rate of evidence accumulation (i.e., *drift rate*), (2) the distance between the two decisional boundaries that quantifies the amount of evidence to facilitate one particular choice alternative (i.e., *decision boundary*), (3) the duration of non-decisional processes, that is, the time taken for processes that are not part of the evidence accumulation process, such as stimulus encoding and motor-response production latency (i.e., *non-decision time*), and (4) possible a priori bias towards one of the two choice alternatives (i.e., *starting point*).

We used the *HDDM toolbox*^39^, an open-source Python software package, to model participants’ RTs and choice accuracy. The HDDM toolbox applies a Bayesian hierarchical framework to estimate the aforementioned four model parameters, in which sampled prior probability distributions of the model parameters are updated based on a likelihood function, formed from the data inputted into the model, to yield posterior probability distributions (Fig. 5a). HDDM uses Markov-Chain Monte Carlo (MCMC) sampling to implement this framework. Specifically, it uses a Gibbs Sampler^92^, via the *PyMC* Python software package^93^, to multiply prior parameter distributions by a likelihood function before normalising to yield posterior parameter distributions capturing joint parameter probability density^94^. As such, we could randomly draw samples that reciprocally constrain participant-level and group-level posterior parameter distributions, yielding more stable parameter estimates^39,95^ (see Fig. 5a). In addition, uncertainty could be directly conveyed using posterior distributions for each estimated parameter, improving model fittings relative to convergence on the most likely value for each parameter observed in traditional DDM approaches^39,96,97^. We further utilised HDDMs as it has been found to be more robust in achieving stable parameter estimates in datasets with fewer trials compared to non-hierarchical DDM approaches^98^.

### Hierarchical drift diffusion model—fitting

To fit HDDMs to participants’ behavioural performance and estimate internal components of perceptual decision formation, we used a process referred to as ‘accuracy-coding’. This fits HDDMs to RT distributions that assume the upper and lower decision boundaries correspond to correct and incorrect choices respectively. Eight HDDM accuracy-coded variants were fit to our behavioural dataset. Seven variants sampled posterior parameter estimates for combinations of drift rate (δ), decision boundary (θ), and non-decision time (τ) for the conditional dependencies (levels) of all three of our predictor (i.e., independent) variables (i.e., sensory condition, stimulus coherence, and age range), whereas one variant was fit to our behavioural dataset that did not allow parameters to vary by our conditional dependencies. Starting point (z) was set as the midpoint between the two decision boundaries for all variants, since stimuli were presented in a pseudorandomised order in the experimental paradigm, thereby considerably reducing the likelihood of an a priori bias towards either choice alternative. In addition, we fixed the trial-to-trial variabilities of each parameter to 0, since previous research has found that these can improve parameter estimates for drift rate (δ), decision boundary (θ), and non-decision time (τ)^99^.

In total, we sampled estimated posterior distributions for a maximum of 12 drift rate (δ), decision boundary (θ), and non-decision time (τ) parameters across all conditional dependencies for the three independent variables (sensory condition: Visual, V trials; Auditory, A trials; Audiovisual, AV trials; stimulus coherence: High Coherence, HC; Low Coherence, LC; age group: Older Adults, OA; Younger Adults; YA, see Supplementary Materials S6 for categorical age split) as follows:

$$\delta ={Y}_{i, j} \sim F\left({{Y}_{i,j} | \delta }_{ HC, LC}^{\begin{array}{l}V, A, AV\\ OA, YA\end{array}}\right)$$

$$\theta ={Y}_{i,j} | \sim F\left({Y}_{i,j} | {\theta }_{ HC, LC}^{\begin{array}{l}V, A. AV\\ OA, YA\end{array}}\right)$$

$$\tau ={Y}_{i,j} | \sim F\left({Y}_{i,j} | {\tau }_{ HC, LC}^{\begin{array}{l}V, A, AV\\ OA, YA\end{array}}\right)$$

where the observed behavioural data (i.e., RTs and binary responses) for participant *i* and trial *j* are distributed by a Wiener joint density distribution (i.e., $$F(\dots )$$; as formulated by^95^) to simultaneously sample individual participant-level and group-level parameters at boundary $${\chi }_{i, j}$$ at time $${T}_{i,j}$$ to complete the prediction of random variable $${Y}_{i,j}$$ (i.e., $${\chi }_{i, j}$$, $${T}_{i,j})$$ across all conditional dependencies.

For each HDDM variant, we ran 5 separate Markov chains with 11,000 samples each. For each chain, the first 1000 were discarded as “burn-in”, and the rest subsampled (“thinned”) by a factor of two, to reduce the autocorrelation within and between Markov chains. This is a conventional approach to MCMC sampling, whereby initial samples in the “burn-in” period are based on the selection of a random starting point, and neighbouring samples therefore likely to be highly correlated. Both issues are likely to provide unreliable posterior distributions for estimated parameters. This left 25,000 remaining samples for each modelling variant, which constituted the probability distributions for each estimated parameter, allowing us to compute individual parameter estimates for participants and condition categories in each variant. To ensure Markov Chain convergence, we computed Gelman-Rubin Ȓ statistics between chains^100^. This compares within-chain and between-chain variance of estimated parameters both for individual participants and group conditions. We verified that all Ȓ statistics fell below 1.02, which suggests reliable convergence between chains.

After assessing modelling convergence, we performed a quantitative comparison of all variants by computing each variant’s associated Deviance Information Criterion (DIC)^101^. The DIC evaluates the trade-off between a modelling variant’s goodness-of-fit and complexity (i.e., number of parameters) when applied to a dataset. We selected the modelling variant with the lowest DIC, which favours the model with the highest likelihood of a goodness-of-fit to the dataset for the least degrees of freedom. Modelling variants with a lower DIC score are to be preferred to those with a higher DIC, indicating the most parsimonious explanation of the dataset. Figure 4b outlines the DICs for each modelling variant. For our HDDM analysis, the modelling variant that best described the data (i.e., the model with the lowest DIC score) was the three-parameter model (Model 8) that sampled drift rate (δ), decision boundary (α), and non-decision time (τ) parameters for each participant (Model 8, $${DIC}_{\begin{array}{c}\alpha \\ \tau \end{array}}^{\delta }=$$ 5719.890). In addition, a difference in DICs greater than 10 indicates substantial evidence that the model with the lower DIC is a better fit^101,102^. Because the difference between the modelling variant with the lowest DIC (Model 8, $${DIC}_{\begin{array}{c}\alpha \\ \tau \end{array}}^{\delta }=$$ 5719.890) and the modelling variant with the second lowest DIC (Model 6, ${DIC}_{\tau }^{\delta }=$ 6184.432) exceeds 10 ($\Delta DIC= -464.542$), we consider this substantial evidence that Model 8 should be considered the most parsimonious account of the behavioural dataset (see Fig. 4b).

Finally, to ensure there were no systematic discrepancies between the empirical dataset and the posited model, we performed a posterior predictive check, simulating a behavioural dataset with the fitted model, and comparing it with the empirical dataset to illustrate that the posited model was a good fit. Figure 4c illustrates that a simulated behavioural dataset based on the best fitted modelling variant (i.e., Model 8, $${DIC}_{\begin{array}{c}\alpha \\ \tau \end{array}}^{\delta }=$$ 5719.890) was consistent with the empirical dataset, and furthermore that the empirical behavioural dataset metrics were within the 90% highest density region (HDR) of the distributions and quantiles of simulated behavioural dataset metrics^103^ (see Supplementary Fig. 1). Therefore, further analyses focused on this modelling variant (i.e., Model 8, $${DIC}_{\begin{array}{c}\alpha \\ \tau \end{array}}^{\delta }=$$ 5719.890).

### Hierarchical drift diffusion model—assessing age-related impacts on multisensory benefits

To further probe multisensory benefits across the adult lifespan, we computed posterior parameter estimation differences between multisensory (i.e., AV trials) versus unisensory (i.e., V or A trials) conditions, collapsing across stimulus coherence levels, and then correlated the subsequent differences with participants’ chronological age using Pearson’s Correlation Coefficients. These were computed to capture age-related trends underlying significant behavioural findings that demonstrated age-related benefits in multisensory decision-making, particularly between AV versus V trials (see “Behavioural results” section).

Furthermore, we compared the multisensory (i.e., AV) drift rates with the optimal combination of the two unisensory (i.e., V + A) drift rates for each individual participant, adopting the following equation proposed by Drugowitsch et al.^20^; see Fig. 7):

$${AV}_{HC}-\sqrt{{V}_{HC}^{2}+{A}_{HC}^{2}}$$

$${AV}_{LC}-\sqrt{{V}_{LC}^{2}+{A}_{LC}^{2} }$$

Here, the difference between the observed drift rates for multisensory (i.e., AV) and combined unisensory (i.e., V + A) trials is calculated for each stimulus coherence trial type (i.e., High Coherence; HC, Low Coherence: LC) to yield drift rate coefficients reflecting the cue-combined accumulation of sensory evidence optimally across sensory conditions and time^20^. Positive (negative) drift rate differences indicate that the multisensory drift rates (do not) supersede the optimal combination of unisensory drift rates.

### Hierarchical drift diffusion model: assessing age-related impacts on the principle of inverse effectiveness

Finally, we sought to assess whether the *principle of inverse effectiveness* (tPoIE) could explain age-related impacts on multisensory decision-making in our HDDM results. According to tPoIE, the magnitude of multisensory benefits increases when the salience of processing individual unimodal stimuli decreases. Hence, the weaker (i.e., less salient) the unimodal stimuli (or the poorer the signal-to-noise ratio), the stronger the multisensory benefit^59–63^. To quantify this, we computed the multisensory benefit for each HDDM parameter as the difference between each individual participant’s multisensory (i.e., MS) and optimal unisensory (i.e., US) drift rate, decision boundary, and non-decision time between stimulus coherence types (i.e., High Coherence; HC, Low Coherence: LC) respectively, as follows:

$${\delta }^{MoIE}= {(\delta }_{LC}^{MS}- {\delta }_{LC}^{US})-({\delta }_{HC}^{MS}- {\delta }_{HC}^{US})$$

$${\theta }^{MoIE}= {(\theta }_{LC}^{MS}- {\theta }_{LC}^{US})-({\theta }_{HC}^{MS}- {\theta }_{HC}^{US})$$

$${\tau }^{MoIE}= {(\tau }_{LC}^{MS}- {\tau }_{LC}^{US})-({\tau }_{HC}^{MS}- {\tau }_{HC}^{US})$$

In which measurements of inverse effectiveness (MoIE) could be computed in the difference between individual participants’ multisensory and optimal unisensory measures; quantified as the highest drift rate (δ), lowest decision boundary (θ), and lowest non-decision time (τ) posterior parameter estimations respectively. To assess if this measurement of inverse effectiveness, and its underlying principle is affected by ageing, we correlated the resultant differences with participants’ chronological age.

### Supplementary Information

Supplementary Figures.

Supplementary Tables.

## Data Availability

Datasets required to reproduce the main analyses can be downloaded from the study’s Open Science Framework repository (https://osf.io/nhk96/).

## Code availability

Source code for main analyses and modelling can be downloaded from the study’s Open Science Framework repository (https://osf.io/nhk96/). Code for reproducing figures is available from the lead author upon reasonable request.
