# Supplementary material for: A drift diffusion model analysis of age-related impact on multisensory decision-making processes
Source: Sci Rep. 2024 Jun 28;14:14895. doi: 10.1038/s41598-024-65549-5 (PMC11213863; doi:10.1038/s41598-024-65549-5)
Supplement: Supplementary file 1 — Supplementary Figures. [file 41598_2024_65549_MOESM1_ESM.docx]

**Supplementary Materials and Figures:**

**Supplementary Materials S1:**

GLMM Analyses – Choice Accuracy – Sensory Condition * Stimulus Coherence Interaction

Our choice accuracy analyses demonstrated significant main effects of sensory condition (χ^2^ = 432.74, df = 2, p < 0.001), in which participants responded more accurately to AV versus V conditions χ^2^ = 79.03, df = 1, p < 0.001), AV versus A conditions (χ^2^ = 437.68, df = 1, p < 0.001), and V versus A conditions (χ^2^ = 145.40, df = 1, p < 0.001), alongside a significant main effect of stimulus coherence, in which participants responded more accurately to HC versus LC trials (χ^2^ = 184.80, df = 1, p < 0.001). A significant two-way interaction was further found between sensory condition and stimulus coherence (χ^2^ = 19.87, df = 2, p < 0.001), in which participants responded more accurately to HC versus LC trials between AV versus A conditions (χ^2^ = 10.89, df = 1, p = 0.001), and V versus A conditions (χ^2^ = 16.91, df = 1, p < 0.001), but not AV versus V conditions (χ^2^ = 0.32, df = 1, p = 0.574). Together, these results suggest larger increases in choice accuracy with the AV trials, relative to V and A trials, with a further decrease with the amount of complementary A stimulus salience, but not for V stimulus salience, benefitting decision accuracy.

*GLMM Analyses – Choice Accuracy - Stimulus Coherence * Chronological Age Interaction*

No significant interaction was found either between stimulus coherence and chronological age (χ^2^ = 3.00, df = 1, p = 0.083), suggesting no age-related differences in categorising sensory conditions of decreasing stimulus salience on decisional accuracy.

*GLMM Analyses – Choice Accuracy – Sensory Condition * Stimulus Coherence * Chronological Age Interaction*

No significant three-way interaction between sensory condition, stimulus coherence, and chronological age (χ^2^ = 1.25, df = 2, p = 0.535). Reduced comparisons reaffirmed this between AV versus V conditions (χ^2^ = 0.58, df = 1, p = 0.447), AV versus A conditions (χ^2^ = 1.23, df = 1, p = 0.268), nor V versus A conditions (χ^2^ = 0.05, df = 1, p = 0.823).

**Supplementary Materials S2:**

GLMM Analyses – Reaction Time – Sensory Condition * Stimulus Coherence Interaction

Our RT analyses demonstrated significant main effects of sensory condition (χ^2^ = 3473.20, df = 2, p < 0.001); in which participants responded faster to AV versus V conditions (χ^2^ = 17.42, df = 1, p < 0.001), AV versus A conditions (χ^2^ = 2560.90, df = 1, p < 0.001), and V versus A conditions (χ^2^ = 2210.70, df = 1, p < 0.001), alongside a significant main effect of stimulus coherence, in which participants responded faster to HC versus LC trials (χ^2^ = 133.71, df = 1, p < 0.001). A significant two-way interaction was further found between sensory condition and stimulus coherence (χ^2^ = 15.15, df = 2, p = 0.001), in which participants responded faster to HC versus LC trials between AV versus A conditions (χ^2^ = 14.02, df = 1, p < 0.001), and between V versus A conditions (χ^2^ = 8.29, df = 1, p = 0.004), but not AV versus V conditions (χ^2^ = 0.740, df = 1, p = 0.3895). Together, these results highlight larger decreases in RTs with AV trials, relative to V and A trials, with a further decrease in RTs with the amount of complementary A evidence salience (i.e., stimulus coherence), but not for V evidence salience, benefitting decision speed.

*GLMM Analyses – Reaction Time – Stimulus Coherence * Chronological Age Interaction*

No significant two-way interaction was found between stimulus coherence and chronological age (χ^2^ = 1.77, df = 1, p = 0.184). This suggests no age-related differences in categorising sensory trials of decreasing stimulus salience (i.e., stimulus coherence) on decisional speed.

*GLMM Analyses – Reaction Time – Sensory Condition * Stimulus Coherence * Chronological Age Interaction*

No significant three-way interaction was reported between sensory condition, chronological age, and stimulus coherence (χ^2^ = 4.20, df = 2, p = 0.376). However, reduced comparisons demonstrated a significant interaction between AV versus V conditions (χ^2^ = 4.53, df = 1, p = 0.033), but not AV versus A conditions (χ^2^ = 0.39, df = 1, p = 0.532), or V versus A conditions (χ^2^ = 0.48, df = 1, p = 0.487).

**Supplementary Materials S3:**

Generalised Linear Mixed-Effects Models (GLMMs) and likelihood-ratio (χ^2^) model comparisons were used to analyse choice accuracy and RTs (using *binomial* logit and *gamma* models respectively) as a function of sensory condition (Visual: V, Auditory: A, Audiovisual: AV trials), stimulus phase coherence (High Coherence: HC/Low Coherence: LC levels respectively, Figure 1b), and categorical age (i.e., age group: Older Adults: OA; N = 100; male = 46, female = 54; mean age = 60.96, SD = 10.35. Younger Adults: YA; N = 112; male = 53, female = 59; mean age = 27.95, SD = 5.82), substituted for chronological age (i.e., decimals), as predictors, as well as all subsequent two-way and three-way analyses

Our main findings demonstrated a significant main effect of categorical age on both RTs and choice accuracy, in which YAs responded faster (χ^2^ = 8.58, df = 1, p = 0.003, Supplementary Figures 2a and 2b) and more accurately (χ^2^ = 21.05, df = 1, p < 0.001, Supplementary Figures 2c and 2d) than OAs across all sensory conditions. In addition, a significant two-way interaction was found between sensory condition and categorical age for RTs (χ^2^ = 120.64, df = 2, p < 0.001, Supplementary Figures 2a and 2b). In particular, we found significant age-related differences in RTs between multisensory (i.e., AV) and unisensory (i.e., V and A) conditions, in which OAs, but not YAs, demonstrated a larger decrease in RTs for AV versus V conditions (χ^2^ = 8.39, df = 1, p = 0.004, Supplementary Figures 3a and 3b), whereas YAs, but not OAs, demonstrated a larger decrease in RTs for AV versus A conditions (χ^2^ = 14.02, df = 1, p < 0.001, Supplementary Figures 4a and 4b), and for V versus A conditions (χ^2^ = 97.49, df = 1, p = 0.003, Supplementary Figures 5a and 5b). Interestingly, a significant two-way interaction was further found between sensory condition and categorical age for choice accuracy (χ^2^ = 9.49, df = 2, p = 0.009). Reduced analyses demonstrated that YAs demonstrated larger increases in choice accuracy between AV versus A conditions (χ^2^ = 4.38, df = 1, p = 0.036, Supplementary Figures 4c and 4d) and between V versus A conditions (χ^2^ = 8.9158, df = 1, p = 0.003, Supplementary Figures 5c and 5d), but not between AV versus V conditions (χ^2^ = 0.57, df = 1, p = 0.452, Supplementary Figures 3c and 3d), relative to OAs.

Overall, our categorical age findings complement the trends we observed for our main chronological age findings. Specially, age-related declines in decision speed (i.e., increased RTs) and accuracy (i.e., decreased proportions of correct responses) were observed between YAs and OAs. In addition, Overall, we observed general age-related declines in decision speed (i.e., increased RTs) and accuracy (i.e., decreased proportions of correct responses). In addition, we saw age-related differences in RTs between multisensory (i.e., AV) versus unisensory (i.e., V and A) conditions. Specifically, OAs tended to display a multisensory benefit in RT differences between AV versus V conditions (i.e., larger AV – V RT differences), irrespective of task difficulty. Coupled with this were no significant age-related impacts on choice accuracy between AV versus V conditions (otherwise observed between AV versus A conditions and V versus A conditions for YAs). These findings strengthen the notion that older adults display preserved, and somewhat enhanced, multisensory RT benefits between AV versus V conditions (i.e., larger AV – V RT difference), particularly for complementary A evidence of decreased task difficulty (i.e., high stimulus coherence), alongside preservations in decisional accuracy.

**Supplementary Materials S4:**

*GLMM Analyses – Choice Accuracy - Stimulus Coherence * Categorical Age Interaction*

No significant interaction was found either between stimulus coherence and categorical age (χ^2^ = 1.85, df = 1, p = 0.174) suggesting no differences between age groups in categorising sensory conditions of decreasing stimulus salience on decisional accuracy.

*GLMM Analyses – Choice Accuracy – Sensory Condition * Stimulus Coherence * Categorical Age Interaction*

No significant three-way interaction was found between sensory condition, stimulus coherence, and categorical age (χ^2^ = 0.96, df = 2, p = 0.618), with reduced comparisons reaffirming this between AV versus V conditions (χ^2^ = 0.52, df = 1, p = 0.470), AV versus A conditions (χ^2^ = 0.91, df = 1, p = 0.341), and V versus A conditions (χ^2^ = 0.119, df = 1, p = 0.913).

**Supplementary Materials S5:**

*GLMM Analyses – Reaction Time - Stimulus Coherence * Categorical Age Interaction*

No significant two-way interaction was found between stimulus coherence and categorical age (χ^2^ = 1.98, df = 1, p = 0.159). This suggests no difference between age groups in categorising sensory trials of decreasing stimulus salience (i.e., stimulus coherence) on decisional speed.

*GLMM Analyses – Reaction Time – Sensory Condition * Stimulus Coherence * Categorical Age Interaction*

No significant three-way interaction was reported between sensory condition, categorical age, and stimulus coherence (χ^2^ = 1.96, df = 2, p = 0.374). This was reaffirmed with reduced comparisons between AV versus V conditions (χ^2^ = 1.99, df = 1, p = 0.158), between AV versus A conditions (χ^2^ = 0.43, df = 1, p = 0.513), and between V versus A conditions (χ^2^ = 0.48, df = 1, p = 0.487).

**Supplementary Materials S6:**

*Hierarchical Drift Diffusion Model – Main Bayesian Hypothesis Testing Results*

Bayesian hierarchical modelling frameworks naturally violate the assumption of independence in its posterior estimation sampling procedure, since group-level and participant level parameter posteriors are simultaneously estimated^39^. Consequently, null-hypothesis significance testing approaches, commonly utilised in frequentist approaches, are not recommended. *Bayesian hypothesis testing* was therefore used to assess strong predictive effects reconciling decisional speed and accuracy in which all levels across sensory conditions, stimulus coherence types, and age groups (i.e., categorical age) are fixed, since our model included an arbitrary split between younger adults (YAs) aged 18-40 and older adults (OAs) aged 40+ (Older Adults: OA; N = 100; male = 46, female = 54; mean age = 60.96, SD = 10.35. Younger Adults: YA; N = 112; male = 53, female = 59; mean age = 27.95, SD = 5.82).

In order to implement Bayesian hypothesis testing to analyse the predictive power of each posterior distribution for each parameter (and its conditional dependencies; see *Supplementary Tables* T1, T2, and T3), we tested hypotheses in which the overlap of separate posterior probability distributions between two parameters (complete with their conditional dependencies) could be compared, returning a value between 0 and 1; with values closer to 0 indicating more prevalent overlaps in posterior probability distributions; and values closer to 1 indicating less prevalent overlaps in posterior probability distributions, depending on the hypothesis tested. Hence, we could assess posterior probability overlaps between parameters (and all their conditional dependencies) from the best fitting HDDM (i.e., lowest DIC; Model 8, ${DIC}_{\begin{aligned} \alpha\\ \tau\end{aligned}}^{\delta}=$ 5719.890) that quantify significant trends in our behavioural measurements (i.e., single-trial RTs and choice accuracy). For example, we could test the hypothesis that younger adults exhibit higher drift rates (i.e., P(δ_YA_ > δ_OA_)), lower decision boundaries (i.e., P(θ_YA_ < θ_OA_)), and lower non-decision times (i.e., P(τ_YA_ < τ_OA_)), relative to older adults, across sensory conditions and levels of stimulus coherence to mechanistically interpret younger adults exhibiting lower RTs, and therefore faster decisional speed, relative to older adults.

To determine the prevalence of true positive results, implicating strong predictive effects in the difference between posterior distributions, we calculated the log posterior odds proportion of a hypothetical proportion corresponding to a false-positive rate of α = 0.05 (i.e., a 95% true-positive threshold, similar to the utilisation of p-values in null-hypothesis significance testing approaches)^104^. Non-overlap proportions greater than the hypothetical log-odds proportion of our false positive rate (which is equal to 2.944) suggest highly predictive effects in the non-overlap of parameter posterior distributions favoured by our hypotheses. Bayesian hypothesis testing of posterior distributions from such bounded HDDMs was able to reconcile decisional speed and accuracy in which all levels across sensory conditions, stimulus coherence types, and age ranges (i.e., categorical age) are fixed. We further performed correlations (using Pearson correlation coefficients) between each participant’s chronological age with their respective HDDM posterior parameter estimations for drift rate (δ), decision boundary (θ), and non-decision time (τ), in order to assess the linear strength and direction of age-related impacts on HDDM parameters.

Our key HDDM findings demonstrated higher drift rates (δ) for YAs, relative to OAs, across all sensory condition and stimulus coherence conditional dependencies (Visual/High Coherence: P($\delta_{YA}> \delta_{OA}$) = > 0.999, log-odds = > 6.660; Visual/Low Coherence: P($\delta_{YA}> \delta_{OA}$) = > 0.999, log-odds = > 6.660; Auditory/High Coherence: P($\delta_{YA}> \delta_{OA}$) = > 0.999, log-odds = > 6.660; Auditory/Low Coherence: P($\delta_{YA}> \delta_{OA}$) = 0.996, log-odds = 5.488; Audiovisual/High Coherence: P($\delta_{YA}> \delta_{OA}$) = > 0.999, log-odds = > 6.660; Audiovisual/Low Coherence: P($\delta_{YA}> \delta_{OA}$) = > 0.999, log-odds = > 6.660, Supplementary Figure 7 *top*), and through observations of significant negative correlations with chronological age across all sensory condition and stimulus coherence conditional dependencies (p = 0.001, Figure 5a). In addition, higher decision boundaries were found for OAs, relative to YAs, only for A/LC and AV/LC conditional dependencies (Visual/High Coherence: P($\theta_{YA}< \theta_{OA}$) = 0.879, log-odds = 1.980; Visual/Low Coherence: P($\theta_{YA}< \theta_{OA}$) = 0.229, log-odds = -1.212; Auditory/High Coherence: P($\theta_{YA}< \theta_{OA}$) = 0.875, log-odds = 1.949; Auditory/Low Coherence: P($\theta_{YA}< \theta_{OA}$) = 0.996, log-odds = 5.488; Audiovisual/High Coherence: P($\theta_{YA}< \theta_{OA}$) = 0.775, log-odds = 1.235; Audiovisual/Low Coherence: P($\theta_{YA}< \theta_{OA}$) = 0.952, log-odds = 2.997, Supplementary Figure 7 *middle*). Correlations with participants’ chronological age reaffirmed these highly predictive results, demonstrating significant positive correlations between decision boundary estimations for AV and A conditions within LC trials (Auditory/Low Coherence: R = 0.26, p = 0.001; Audiovisual/Low Coherence: R = 0.18, p = 0.001, Figure 5b), implying an increased response caution for OAs when processing AV and A stimuli of increased task difficulty.

Between AV versus V trials, drift rate estimations were higher for all conditional dependencies except for YAs within HC trial types (Older Adults/High Coherence: P($\delta_{AV}> \delta_{V}$) = 0.996, log-odds = 5.538; Older Adults/Low Coherence: P($\delta_{AV}> \delta_{V}$) = 0.986, log-odds = 4.278; Younger Adults/High Coherence: P($\delta_{AV}> \delta_{V}$) = 0.948, log-odds = 2.902; Younger Adults/Low Coherence: P($\delta_{AV}> \delta_{V}$) = 0.997, log-odds = 6.570, Supplementary Figure 7 *top*). These results are consistent with the significantly shorter RTs and higher proportions of correct responses observed for AV compared to V conditions, as well as the significant interactions between reduced sensory condition (i.e., AV versus V conditions) and stimulus coherence and age group conditions respectively, suggesting that the rate of visual sensory evidence accumulation is enhanced when presented simultaneously with complementary auditory information (except in YAs with decreased trial difficulty. Interestingly, correlations with chronological age revealed a significant positive correlation with AV – V drift rate differences within HC trials alone (Audiovisual – Visual/High Coherence: R = 0.15, p = 0.025; Audiovisual – Visual/Low Coherence: R = -0.014, p = 0.840, Figure 6a *top*). Together, this implies that older adults display enhanced multisensory integration, through RT benefits towards perceptual decision formation between AV versus V conditions of decreased task difficulty (i.e., HC trials), as highlighted by the significantly greater RT difference exhibited in our behavioural results, which remains preserved between AV versus V conditions of increased task difficulty (i.e., LC trials).

For decision boundary (θ) estimations, we found (1) higher θs for AV versus V trials for OAs within LC trials, and (2) higher θs for AV versus V trials for YAs within HC trials (Older Adults/High Coherence: P($\theta_{AV}> \theta_{V}$) = 0.925, log-odds = 2.515; Older Adults/Low Coherence: P($\theta_{AV}> \theta_{V}$) = 0.999, log-odds = 6.660; Younger Adults/High Coherence: P($\theta_{AV}> \theta_{V}$) = 0.966, log-odds = 3.355; Younger Adults/Low Coherence: P($\theta_{AV}> \theta_{V}$) = 0.751, log-odds = 1.106, *Supplementary Figure* 7 *middle*). Correlations with chronological age, however, uncovered a significant positive correlation between AV versus V conditions within LC trials alone (Audiovisual – Visual/High Coherence: R = -0.018, p = 0.790; Audiovisual – Visual/Low Coherence: R = 0.15, p = 0.029; Audiovisual – Auditory/High Coherence: R = -0.005, p = 0.940; Audiovisual – Auditory/Low Coherence: R = 0.003, p = 0.960; Visual – Auditory/High Coherence: R = 0.01, p = 0.880; Visual – Auditory/Low Coherence: R = -0.13, p = 0.061, Figure 6b). These results suggest that OAs display increased caution in decision policy for AV versus V conditions when complementary A evidence is consolidated with increased task difficulty (i.e., LC trials). Given we observed in our behavioural results that OAs displayed a greater multisensory benefit towards RTs between AV versus V conditions, coupled with (1) no significant differences in choice accuracy between AV versus V conditions, and (2) a significant reduced three-way interaction suggesting such multisensory benefits are impacted within LC trial types, OAs are implicated to display increase caution in choice responses when complementary sensory evidence is more difficult to categorise between AV versus V conditions, thus preserving RT benefits.

Overall, we observed general age-related decreases in drift rate between OAs versus YAs, coupled with general age-related decreases as a function of chronological age, across all sensory conditions and stimulus coherence types, thus implying OAs accumulated sensory evidence more slowly than YAs. A comparison of drift rates between AV and V conditions within OA and YA age ranges, however, demonstrated that OAs consistently exhibited greater increases in drift rate for AV versus V conditions irrespective of task difficulty (i.e., task difficulty). Interestingly, YAs did not show this drift rate trend for HC trial types, suggesting that when the task is less difficulty, the consolidation of complementary A information does not benefit perceptual decision formation. This was further captured by an age-related AV – V difference increase for HC trial types, but no age-related AV – V differences for LC trial types. Coupled with this were higher decision boundaries for AV versus V trial types of increased trial difficulty (i.e., LC trial types) for OAs versus YAs, demonstrating that for general multisensory processing and specific unisensory processing, OAs had a higher response caution in decision policy, in which more sensory evidence needed to be accumulated to facilitate decisions.

**Supplementary Materials S7:**

*Hierarchical Drift Diffusion Modelling Results - Age Group*

We found higher drift rates (δ) for YAs versus OAs across all sensory condition and stimulus coherence conditional dependencies (Visual/High Coherence: P($\delta_{YA}> \delta_{OA}$) = > 0.999, log-odds = > 6.660; Visual/Low Coherence: P($\delta_{YA}> \delta_{OA}$) = > 0.999, log-odds = > 6.660; Auditory/High Coherence: P($\delta_{YA}> \delta_{OA}$) = > 0.999, log-odds = > 6.660; Auditory/Low Coherence: P($\delta_{YA}> \delta_{OA}$) = 0.996, log-odds = 5.488; Audiovisual/High Coherence: P($\delta_{YA}> \delta_{OA}$) = > 0.999, log-odds = > 6.660; Audiovisual/Low Coherence: P($\delta_{YA}> \delta_{OA}$) = > 0.999, log-odds = > 6.660, Supplementary Figure 7 *top*). We reaffirm these prominent age-related declines in drift rates through observations of significant negative correlations with chronological age across all sensory condition and stimulus coherence conditional dependencies (p = 0.001, Figure 5a). These results are consistent with the significantly higher RTs and lower proportions of correct responses observed for OAs across all sensory conditions. Since the drift rate parameter captures the average rate of sensory evidence accumulation, these results implicate that older adults accumulate sensory evidence at a slower rate than younger adults.

Decision boundary (θ) estimations were higher for OAs versus YAs, but only for AV and A trials within LC trials (Visual/High Coherence: P($\theta_{YA}< \theta_{OA}$) = 0.879, log-odds = 1.980; Visual/Low Coherence: P($\theta_{YA}< \theta_{OA}$) = 0.229, log-odds = -1.212; Auditory/High Coherence: P($\theta_{YA}< \theta_{OA}$) = 0.875, log-odds = 1.949; Auditory/Low Coherence: P($\theta_{YA}< \theta_{OA}$) = 0.996, log-odds = 5.488; Audiovisual/High Coherence: P($\theta_{YA}< \theta_{OA}$) = 0.775, log-odds = 1.235; Audiovisual/Low Coherence: P($\theta_{YA}< \theta_{OA}$) = 0.952, log-odds = 2.997, Supplementary Figure 7 *middle*). Correlations with participants’ chronological age reaffirmed these highly predictive results, demonstrating significant positive correlations between decision boundary estimations for AV and A trials within LC trials (Auditory/Low Coherence: R = 0.26, p = 0.001; Audiovisual/Low Coherence: R = 0.18, p = 0.001, Figure 6b *bottom*), implying an increased response caution for OAs when processing AV and A stimuli of decreased salience. A significant positive correlation was observed for decision boundary estimations for V within HC trials (Figure 5b; Visual/High Coherence: R = 0.17, p = 0.011, Figure 5b), but was not reaffirmed by the hypothesis assessing categorical age-related differences.

Non-decision time (τ) estimations were: (1) higher for OAs versus YAs only for V trials, and (2) lower for OAs versus YAs only for A within LC trials respectively (Visual/High Coherence: P($\tau_{YA}< \tau_{OA}$) = 0.352, log-odds = -0.609; Visual/Low Coherence: P($\tau_{YA}< \tau_{OA}$) = 0.979, log-odds = 3.846; Auditory/High Coherence: P($\tau_{YA}< \tau_{OA}$) = 0.076, log-odds = -2.495; Auditory/Low Coherence: P($\tau_{YA}< \tau_{OA}$) = 0.006, log-odds = -5.158; Audiovisual/High Coherence = 0.374, log-odds = -0.516; Audiovisual/Low Coherence = 0.156, log-odds = -1.686, Supplementary Figure 7 *bottom*). However, a significant positive correlation with chronological age was found for (1) higher non-decision time estimations for OAs versus YAs for V trials within LC trials (Visual/Low Coherence: R = 0.25, p = 0.001; Auditory/Low Coherence: R = -0.11, p = 0.110, Figure 5c). Interestingly, no prevalent differences were found between OAs and YAs for AV trial types, which was reaffirmed through non-significant correlations with chronological age (Audiovisual/High Coherence: R = 0.083, p = 0.230; Audiovisual/Low Coherence: R = 0.057, p = 0.410, Figure 5c). Coupled with the different trajectories of non-decisional processing between OAs and YAs, this result underlies the comparative multisensory benefits exhibited between multisensory (i.e., AV) and unisensory (i.e., V and A) trials on RTs, and YAs benefit more from non-decisional processing speed from complementary V evidence alone.

*Sensory Condition*

We found higher drift rates (δ) for AV versus A conditions (Older Adults/High Coherence: P($\delta_{AV}> \delta_{A}$) = > 0.999, log-odds = > 6.660; Older Adults/Low Coherence: P($\delta_{AV}> \delta_{A}$) = > 0.999; Younger Adults/High Coherence: P($\delta_{AV}> \delta_{A}$) = > 0.999, log-odds = > 6.660; Younger Adults/Low Coherence: P($\delta_{AV}> \delta_{A}$) = > 0.999, log-odds = > 6.660) and V versus A trials (Older Adults/High Coherence: P($\delta_{V}> \delta_{A}$) = > 0.999, log-odds = >6.660; Older Adults/Low Coherence: P($\delta_{V}> \delta_{A}$) = 0.966, log-odds = 3.343; Younger Adults/High Coherence: P($\delta_{V}> \delta_{A}$) = > 0.999, log-odds = > 6.660; Younger Adults/Low Coherence: P($\delta_{V}> \delta_{A}$) = > 0.999, log-odds = > 6.660, Supplementary Figure 7 *top*) across all conditional dependencies. However, correlations with chronological age only reaffirmed a significant negative correlation between V versus A conditions within HC trials (Audiovisual – Auditory/High Coherence: R = -0.082, p = 0.230; Audiovisual – Auditory/Low Coherence: R = -0.096, p = 0.160; Visual – Auditory/High Coherence: R = -0.17, p = 0.015; Visual – Auditory/Low Coherence: R = -0.072, p = 0.300, Figure 8a). Taken together, these results suggest a prominent trend of increased A evidence accumulation as a function of age when stimulus salience decreases alone, reconciling the discrepancies between categorical age hypothesis testing and chronological age correlation analyses.

Between AV versus V conditions, drift rate estimations were higher for all conditional dependencies except for YAs within HC trial types (Older Adults/High Coherence: P($\delta_{AV}> \delta_{V}$) = 0.996, log-odds = 5.538; Older Adults/Low Coherence: P($\delta_{AV}> \delta_{V}$) = 0.986, log-odds = 4.278; Younger Adults/High Coherence: P($\delta_{AV}> \delta_{V}$) = 0.948, log-odds = 2.902; Younger Adults/Low Coherence: P($\delta_{AV}> \delta_{V}$) = 0.997, log-odds = 6.570, Supplementary Figure 7 *top*). These results are consistent with the significantly shorter RTs and higher proportions of correct responses observed for AV compared to V conditions, as well as the significant interactions between reduced sensory condition (i.e., AV versus V conditions) and stimulus coherence and age range conditions respectively, suggesting that the rate of visual sensory evidence accumulation is enhanced when presented simultaneously with complementary auditory information (except in YAs with high evidence salience). Interestingly, correlations with chronological age revealed a significant positive correlation with AV – V drift rate differences within HC trials alone (Audiovisual – Visual/High Coherence: R = 0.15, p = 0.025; Audiovisual – Visual/Low Coherence: R = -0.014, p = 0.840, Figure 6a). Together, this implies that older adults display enhanced multisensory integration, through RT benefits towards perceptual decision formation between AV versus V conditions of high evidence salience (i.e., HC trials), as highlighted by the significantly greater RT difference exhibited in our behavioural results, which remains preserved between AV versus V conditions of low evidence salience (i.e., LC trials).

For decision boundary (θ) estimations, we found (1) higher θs for AV versus V conditions for OAs within LC trials, and (2) higher θs for AV versus V conditions for YAs within HC trials (Older Adults/High Coherence: P($\theta_{AV}> \theta_{V}$) = 0.925, log-odds = 2.515; Older Adults/Low Coherence: P($\theta_{AV}> \theta_{V}$) = 0.999, log-odds = 6.660; Younger Adults/High Coherence: P($\theta_{AV}> \theta_{V}$) = 0.966, log-odds = 3.355; Younger Adults/Low Coherence: P($\theta_{AV}> \theta_{V}$) = 0.751, log-odds = 1.106, Supplementary Figure 7 *middle*). In comparison, no prevalent differences in decision boundary estimates were found between AV versus A conditions across any conditional dependencies (Older Adults/High Coherence: P($\theta_{AV}> \theta_{A}$) = 0.537, log-odds = 0.150; Older Adults/Low Coherence: P($\theta_{AV}> \theta_{A}$) = 0.135, log-odds = -1.855; Younger Adults/High Coherence: P($\theta_{AV}> \theta_{A}$) = 0.673, log-odds = 0.721; Younger Adults/Low Coherence: P($\theta_{AV}> \theta_{A}$) = 0.431, log-odds = -0.277, Supplementary Figure 7 *middle*), coupled with higher decision boundary estimates for V versus A conditions only for OAs within LC trials (Older Adults/High Coherence: P($\theta_{V}> \theta_{A}$) = 0.083, log-odds = -2.401; Older Adults/Low Coherence: P($\theta_{V}> \theta_{A}$) = > 0.999, log-odds = > 6.660; Younger Adults/High Coherence: P($\theta_{V}> \theta_{A}$) = 0.071, log-odds = -2.567; Younger Adults/Low Coherence: P($\theta_{V}> \theta_{A}$) = 0.191, log-odds = -1.440, Supplementary Figure 7 *middle*). Correlations with chronological age, however, uncovered a significant positive correlation between AV versus V trials within LC trials alone (Audiovisual – Visual/High Coherence: R = -0.018, p = 0.790; Audiovisual – Visual/Low Coherence: R = 0.15, p = 0.029; Audiovisual – Auditory/High Coherence: R = -0.005, p = 0.940; Audiovisual – Auditory/Low Coherence: R = 0.003, p = 0.960; Visual – Auditory/High Coherence: R = 0.01, p = 0.880; Visual – Auditory/Low Coherence: R = -0.13, p = 0.061, Figure 6b *bottom*). These results suggest that OAs display increased caution in decision policy for AV versus V conditions when complementary A evidence is of decreased stimulus salience (i.e., LC trials). Given we observed in our behavioural results that OAs displayed a greater multisensory benefit towards RTs between AV versus V trials, coupled with (1) no significant differences in choice accuracy between AV versus V trials, and (2) a significant reduced three-way interaction suggesting such multisensory benefits are impacted within LC trial types, OAs are implicated to display increase caution in choice responses when complementary sensory evidence is more difficult to categorise between AV versus V trials, thus preserving RT benefits.

For non-decision time (τ) estimations, we found (1) lower τs for AV versus V conditions for OAs in LC trials (Older Adults/High Coherence: P($\tau_{AV}< \tau_{V}$) = 0.643, log-odds = 0.587; Older Adults/Low Coherence: P($\tau_{AV}< \tau_{V}$) = 0.986, log-odds = 4.278; Younger Adults/High Coherence: P($\tau_{AV}< \tau_{V}$) = 0.674, log-odds = 0.725; Younger Adults/Low Coherence: P($\tau_{AV}< \tau_{V}$) = 0.224, log-odds = -1.242), (2) lower τs for AV versus A conditions for YAs across HC/LC trials(Older Adults/High Coherence: P($\tau_{AV}< \tau_{A}$) = 0.882, log-odds = 2.010; Older Adults/Low Coherence: P($\tau_{AV}< \tau_{A}$) = 0.779, log-odds = 1.379 ; Younger Adults/High Coherence: P($\tau_{AV}< \tau_{A}$) = 0.992, log-odds = 4.877; Younger Adults/Low Coherence: P($\tau_{AV}< \tau_{A}$) = 0.995, log-odds = 5.246, Supplementary Figure 7 *bottom*), and (3) lower τs for V versus A conditions for YAs across HC/LC trials (Older Adults/High Coherence: P($\tau_{V}< \tau_{A}$) = 0.796, log-odds = 1.362; Older Adults/Low Coherence: P($\tau_{V}< \tau_{A}$) = 0.087, log-odds = 2.346; Younger Adults/High Coherence: P($\tau_{V}< \tau_{A}$) = 0.978, log-odds = 3.782; Younger Adults/Low Coherence: P($\tau_{V}< \tau_{A}$) = 0.999, log-odds = 6.660, Supplementary Figure 7 *bottom*). Interestingly, these results suggest that despite increased response caution in decision policy, OAs had a lower duration for encoding AV versus V information of decreased stimulus salience, whereas YAs saw a further benefit encoding AV versus A information regardless of stimulus salience, enhancing benefits towards RTs and choice accuracy overall. This trend can be reaffirmed given YAs had a lower duration for encoding V versus A conditions (regardless of stimulus salience). Correlations with chronological age, however, did not yield any significant findings, suggesting the highlighted predictive non-decision time results were not prominently impacted as a function of age (Audiovisual – Visual/High Coherence: R = 0.037, p = 0.590; Audiovisual – Visual/Low Coherence: R = -0.034, p = 0.620; Audiovisual – Auditory/High Coherence: R = -0.071, p = 0.300; Audiovisual – Auditory/Low Coherence: R = -0.062, p = 0.370; Audiovisual – Auditory/Low Coherence: R = -0.062, p = 0.370; Visual – Auditory/High Coherence: R = -0.095, p = 0.170; Visual – Auditory/Low Coherence: R = -0.040, p = 0.560, Figure 6c and Supplementary Figure 8c).

*Stimulus Coherence*

We found higher drift rate (δ) estimations for HC versus LC trials across all sensory conditions and age range conditions (Older Adults/Visual: P($\delta_{HC}> \delta_{LC}$) = 0.999, log-odds = 6.660; Younger Adults/Visual: P($\delta_{HC}> \delta_{LC}$) = > 0.999, log-odds = > 6.660; Older Adults/Auditory: P($\delta_{HC}> \delta_{LC}$) = 0.957, log-odds = 3.091; Younger Adults/Auditory: P(($\delta_{HC}> \delta_{LC}$) = 0.998, log-odds = 6.064; Older Adults/Audiovisual: P($\delta_{HC}> \delta_{LC}$) = > 0.999, log-odds = > 6.660; Younger Adults/Audiovisual: P($\delta_{HC}> \delta_{LC}$) = > 0.999, log-odds = > 6.660, Supplementary Figure 7 *top*). These findings are consistent with behavioural results illustrating significantly lower RTs for HC compared to LC trials, implicating decreases in the rate of sensory evidence accumulation with decreasing stimulus salience (i.e., increased task difficulty), and vice versa.

For decision boundary (θ) estimations, we found higher estimations for HC versus LC trials for (a) OAs in V conditions, and (b) YAs in AV conditions (Older Adults/Visual: P($\theta_{HC}> \theta_{LC}$) = 0.998, log-odds = 6.213; Younger Adults/Visual: P($\delta_{HC}> \delta_{LC}$) = 0.826, log-odds = 1.556; Older Adults/Auditory: P($\theta_{HC}> \theta_{LC}$) = 0.529, log-odds = 0.114; Younger Adults/ Auditory : P($\theta_{HC}> \theta_{LC}$) = 0.944, log-odds = 2.832; Older Adults/Audiovisual: P($\theta_{HC}> \theta_{LC}$) = 0.892, log-odds = 2.113; Younger Adults/Audiovisual: P($\theta_{HC}> \theta_{LC}$) = 0.984, log-odds = 4.137, Supplementary Figure 7 *middle*). These findings indicate increased response caution was not a factor within AV or A conditions for older adults, yet was a factor in AV conditions for younger adults, and is consistent with general increases in choice accuracy observed for younger adults, but overall lack of significant findings for choice accuracy between OAs versus YAs with stimulus coherence.

For non-decision time (τ) estimations, we found lower estimations for HC versus LC trials for OAs and V trial types only (Older Adults/Visual: P($\tau_{HC}< \tau_{LC}$) = 0.958, log-odds = 3.126; Younger Adults/Visual: P($\tau_{HC}< \tau_{LC}$) = 0.253, log-odds = -1.053; Older Adults/Auditory: P($\tau_{HC}< \tau_{LC}$) = 0.313, log-odds = -0.788; Younger Adults/Auditory: P($\tau_{HC}< \tau_{LC}$) = 0.746, log-odds = 1.078; Older Adults/Audiovisual: P($\tau_{HC}< \tau_{LC}$) = 0.445, log-odds = -0.223; Younger Adults/Audiovisual: P($\tau_{HC}< \tau_{LC}$) = 0.708, log-odds = 0.885, Supplementary Figure 7 *bottom*). These findings indicate that stimulus salience (i.e., task difficulty) was modality-specific in decreasing the duration of non-decisional processes for older adults with visual information only.

[104] Ince, R. A., Paton, A. T., Kay, J. W. & Schyns, P. G. Bayesian inference of population prevalence. *Elife* **10**, e62461 (2021)


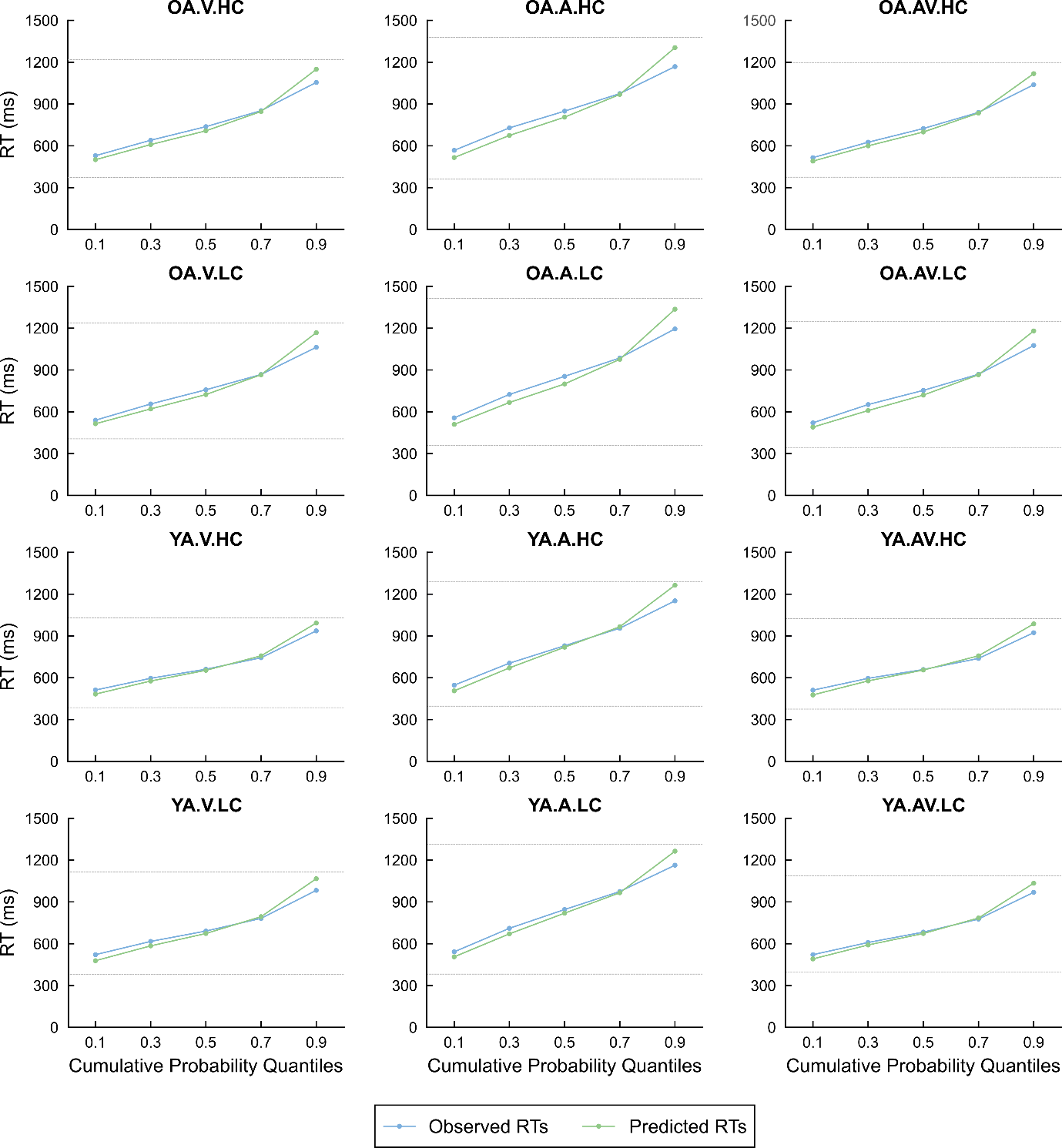


**Supplementary Figure 1: HDDM - Posterior Predictive Checks (Highest Density Regions).** Posterior predictive checks of the best fitting HDDM for the behavioural dataset. Modelling fit to the behavioural dataset was assessed using a cumulative quantile-probability plot, showing quantiles of RT distributions split across all conditional dependencies. Cumulative probability quantiles are plotted along the x-axis for observed RTs (blue) and predicted RTs (green), i.e. simulated RTs from HDDM posterior predictive estimates. Dashed grey lines on the y-axis denote the credible intervals representing the Highest (posterior) Density Region (HDR) at a 90% confidence level.

**
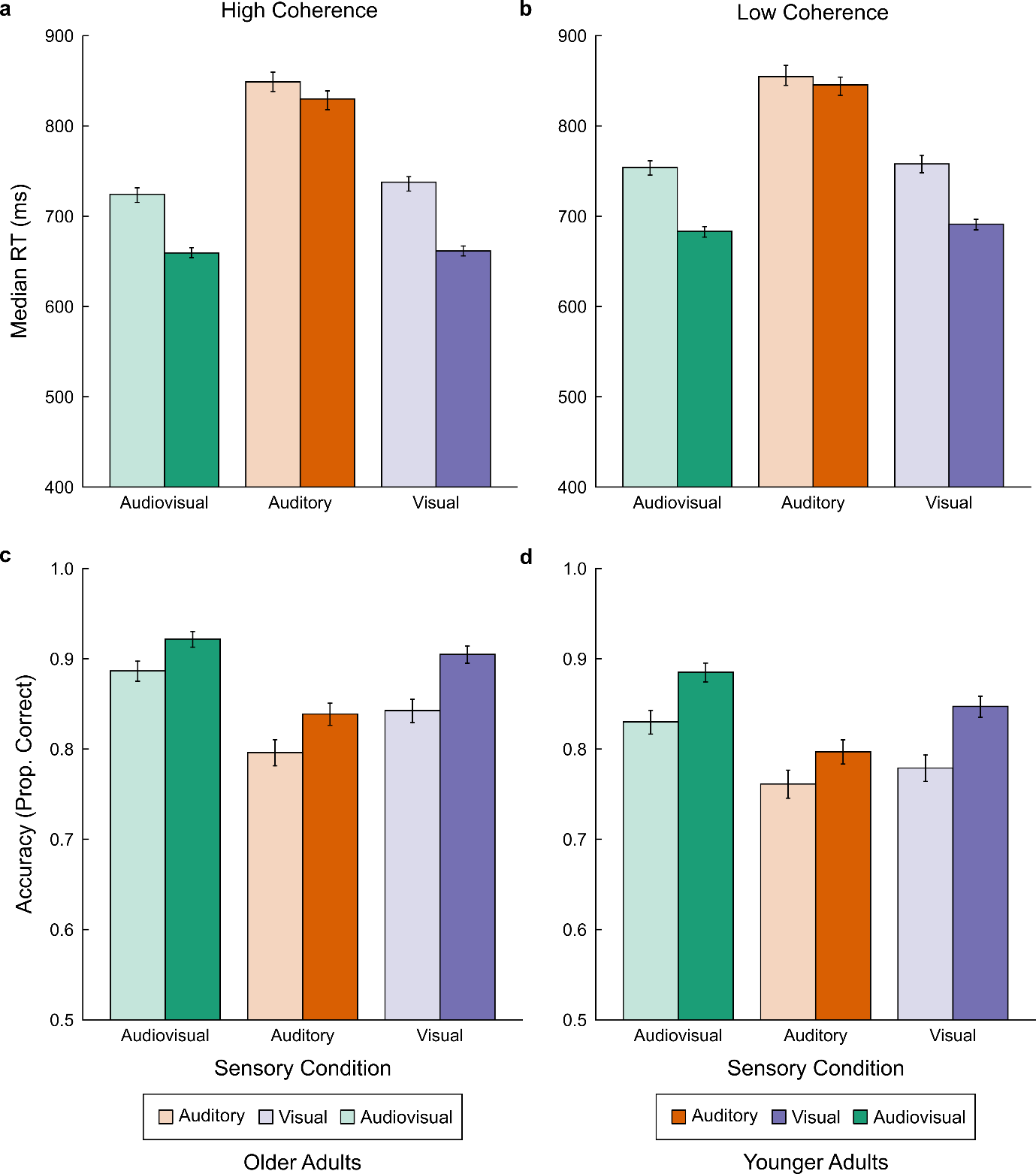
**

**Supplementary Figure 2: Behavioural Performance. a**, **b**, Median RTs (*top*) and **c**, **d**, choice accuracy (proportion of correct responses; *bottom*) for High Coherence (*left*) and Low Coherence (*right*) trials, across levels of age group (Older Adults/Younger Adults), as a function of Audiovisual (green), Auditory (orange), and Visual (purple) sensory conditions. Error bars indicate 95% Confidence Intervals (CIs), which were calculated using 1000 bootstrapping random sampling interactions to estimate the distribution of group average performance measurements.


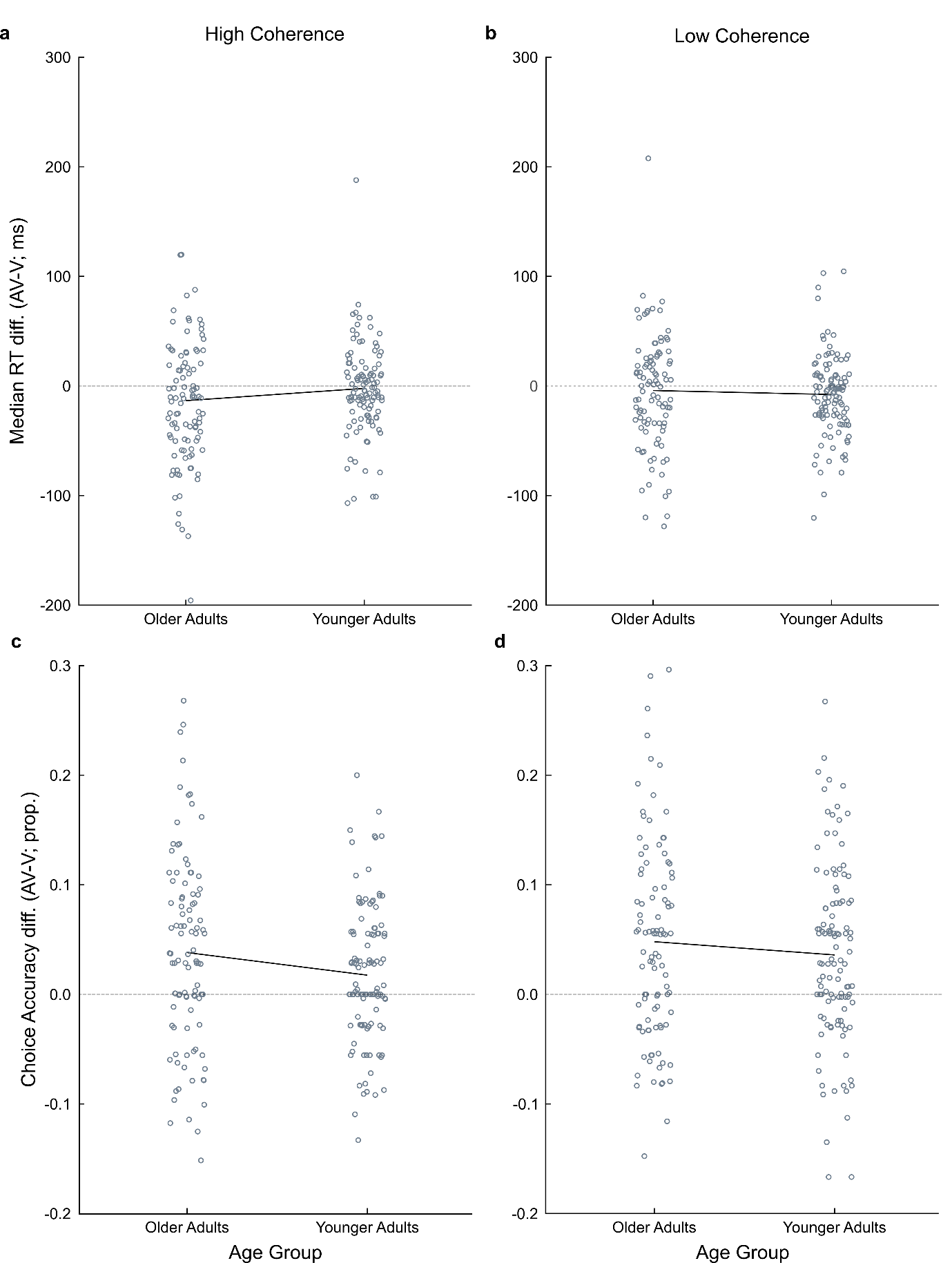


**Supplementary Figure 3: AV – V Behavioural Performance Differences.** Individual participant behavioural performance differences between AV versus V conditions (AV – V). **a**, **b**, AV versus V median RT differences (AV – V median RT diff.) and **c**, **d**, AV versus V )AV – V choice accuracy diff.) proportion correct differences for Older Adults and Younger Adults across High Coherence (*left*) and Low Coherence (right) trials. Solid black lines denote the group averages (calculated across N = 212 participants), and dashed grey lines denote the boundaries for no behavioural performance differences.


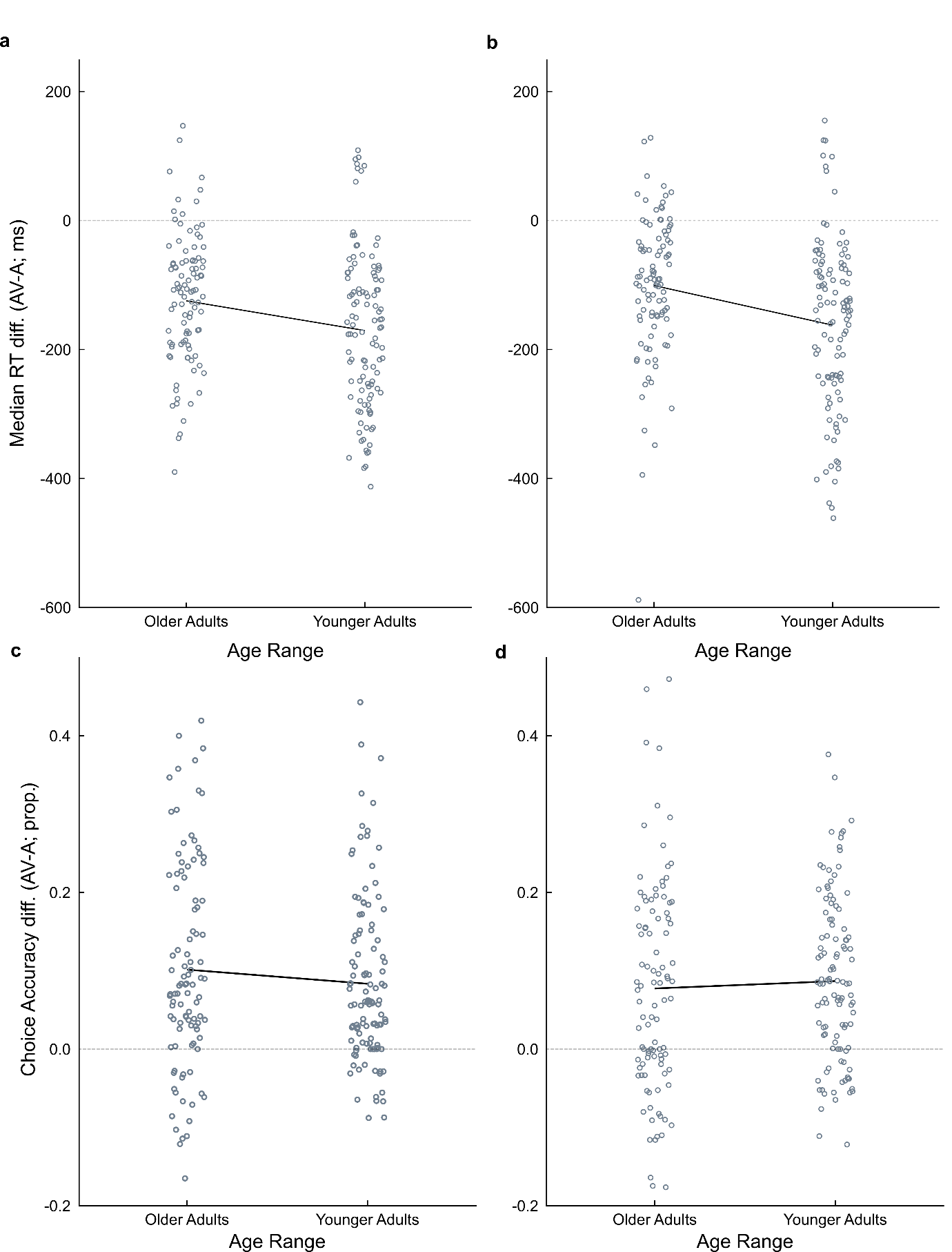


**Supplementary Figure 4: AV – A Performance Differences.** Individual participant (proportion correct) performance differences between **a**, **b**, AV and A median RT difference (V – A) sensory trial types (*top*), and **c**, **d**, AV and A proportion correct differences (V – A) sensory conditions (*bottom*) for Older Adults and Younger Adults across High Coherence (*left*) and Low Coherence (*right*) trial types. Solid black lines denote the group averages (calculated across N = 212 participants), and dashed grey lines denote the boundaries for no behavioural performance differences.


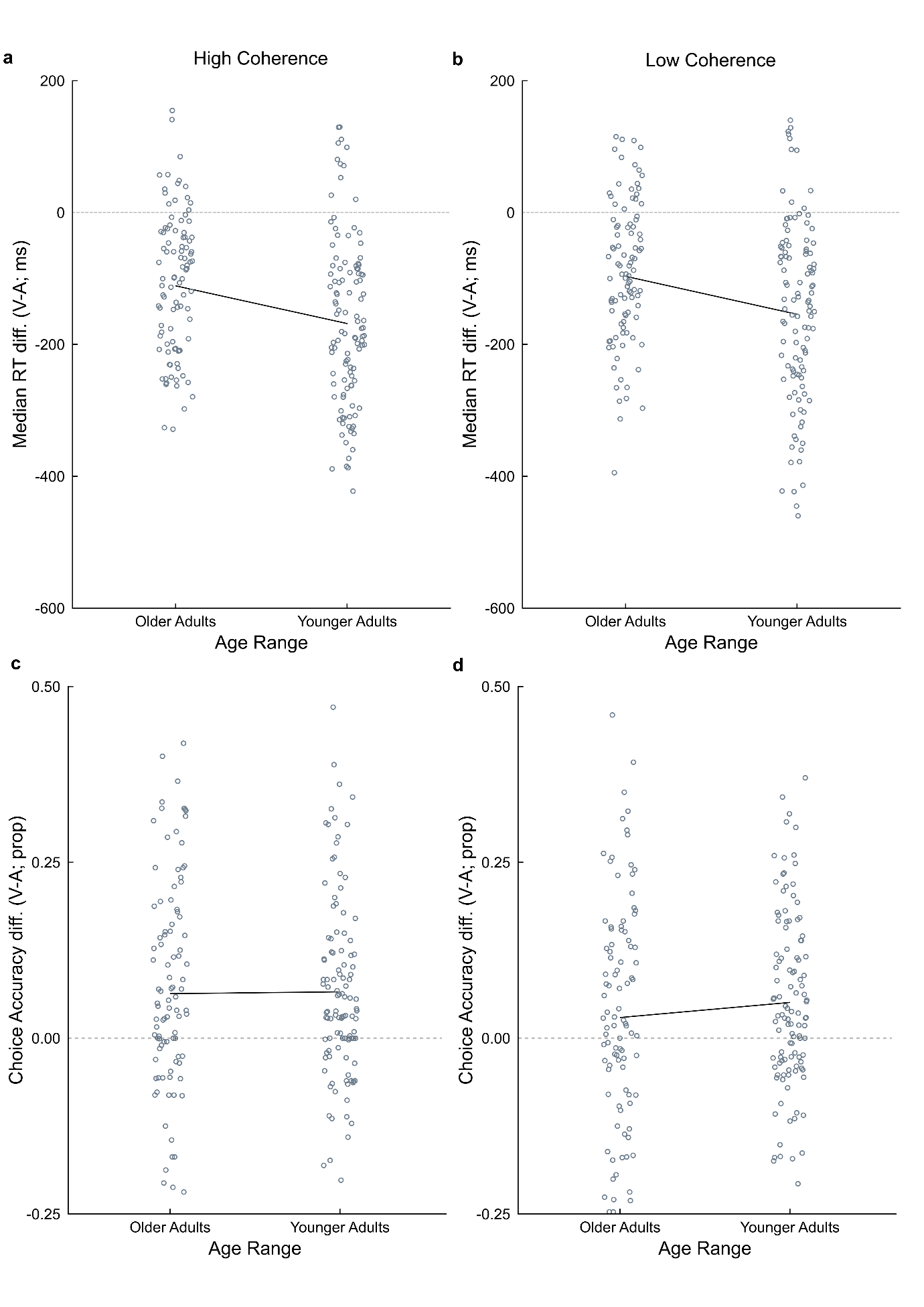


**Supplementary Figure 5: V – A Performance Differences.** Individual participant (proportion correct) performance differences between **a**, **b**, V and A median RT difference (V – A) sensory conditions (*top*), and **c**, **d**, V and A proportion correct differences (V – A) sensory trial types (*bottom*) for Older Adults and Younger Adults across High Coherence (*left*) and Low Coherence (*right*) trials. Solid black lines denote the group averages (calculated across N = 212 participants), and dashed grey lines denote the boundaries for no behavioural performance differences.


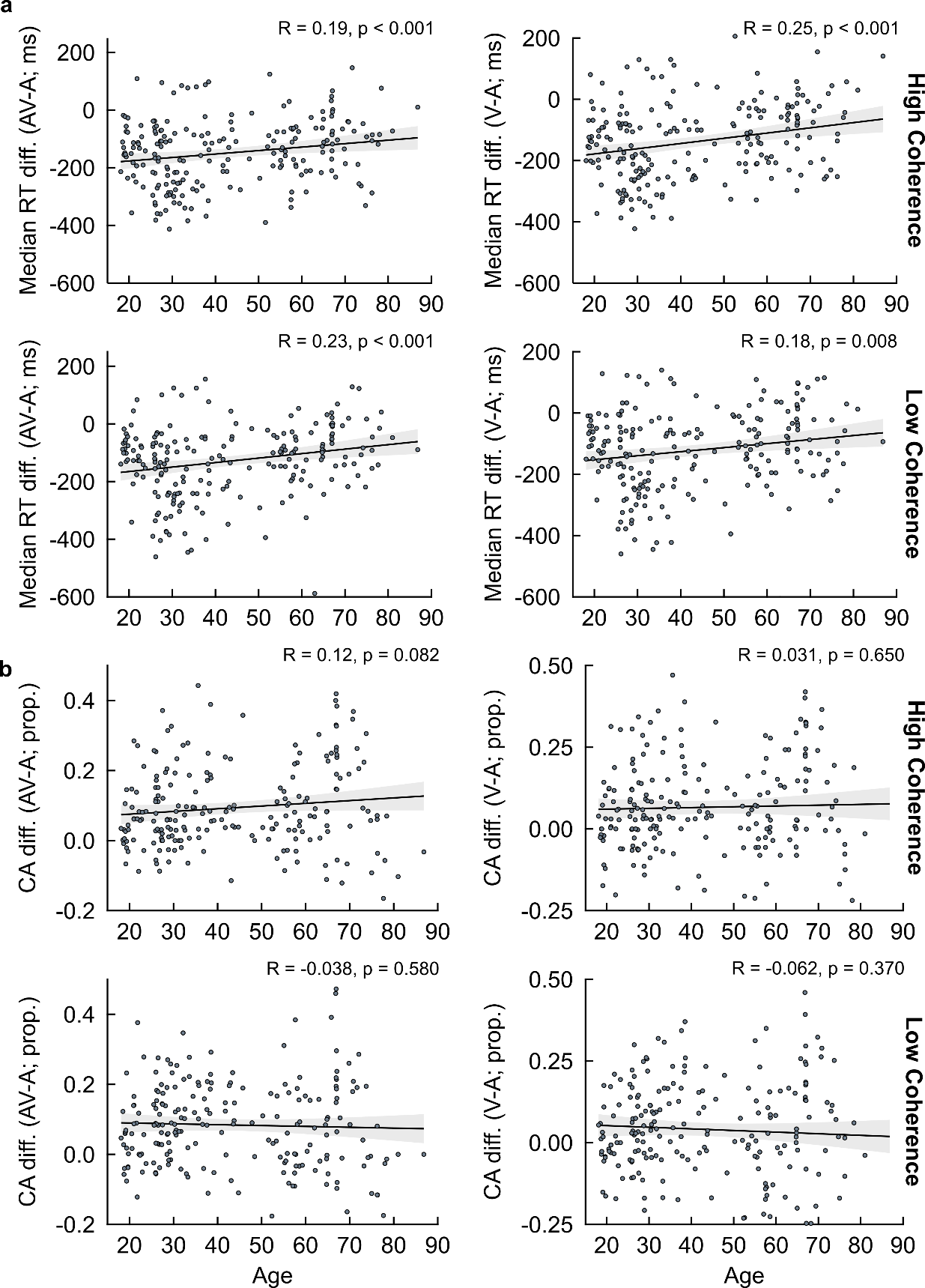


**Supplementary Figure 6: (A)V versus A Behavioural Performance Differences - Correlations with Chronological Age.** Individual Pearson’s correlations of age with **a**, median RT differences and **b**, choice accuracy (proportion of correct responses) differences between AV and A conditions (*left*), and between and V and A conditions (*right*), across the two levels of stimulus coherence (high/low coherence). Pearson Correlation Coefficients (R) and p-values are shown for each correlation. Shaded regions indicate 95% Confidence Intervals (CIs).


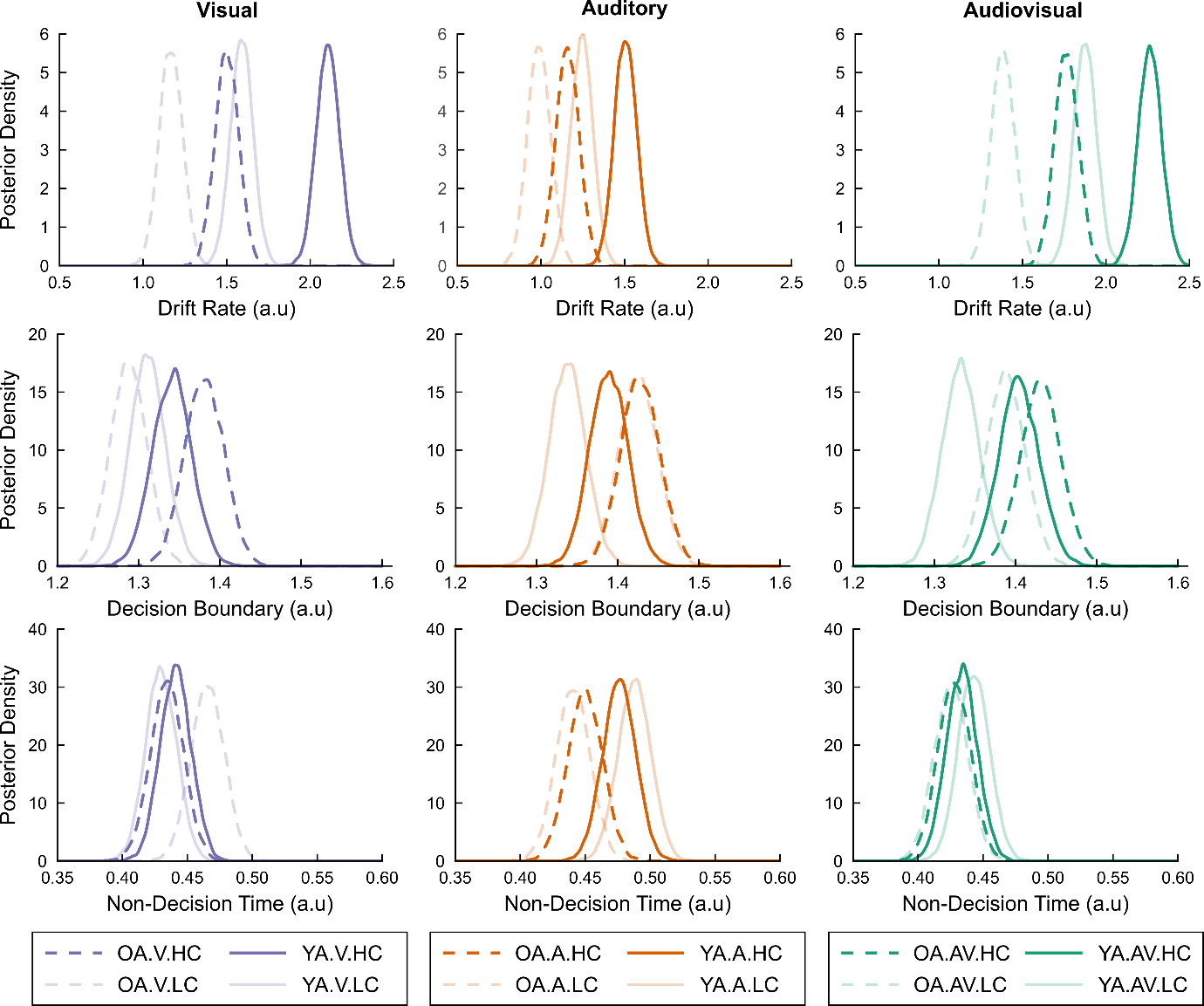


**Figure 7: HDDM Posterior Parameter Estimations – Age Range Conditional Dependencies.** Group posterior density distributions illustrating ageing conditional dependencies for **a**, drift rate (δ; *top*), **b**, decision boundary (θ; *middle*), and **c**, non-decision time (τ; *bottom*) parameters across sensory conditions (Visual, V; Auditory; A; Audiovisual; A) and stimulus coherence (High Coherence, HC; Low Coherence; LC) conditional dependencies All posterior density distributions are derived from the behavioural HDDM, including N = 212 participants and 41765 trials.


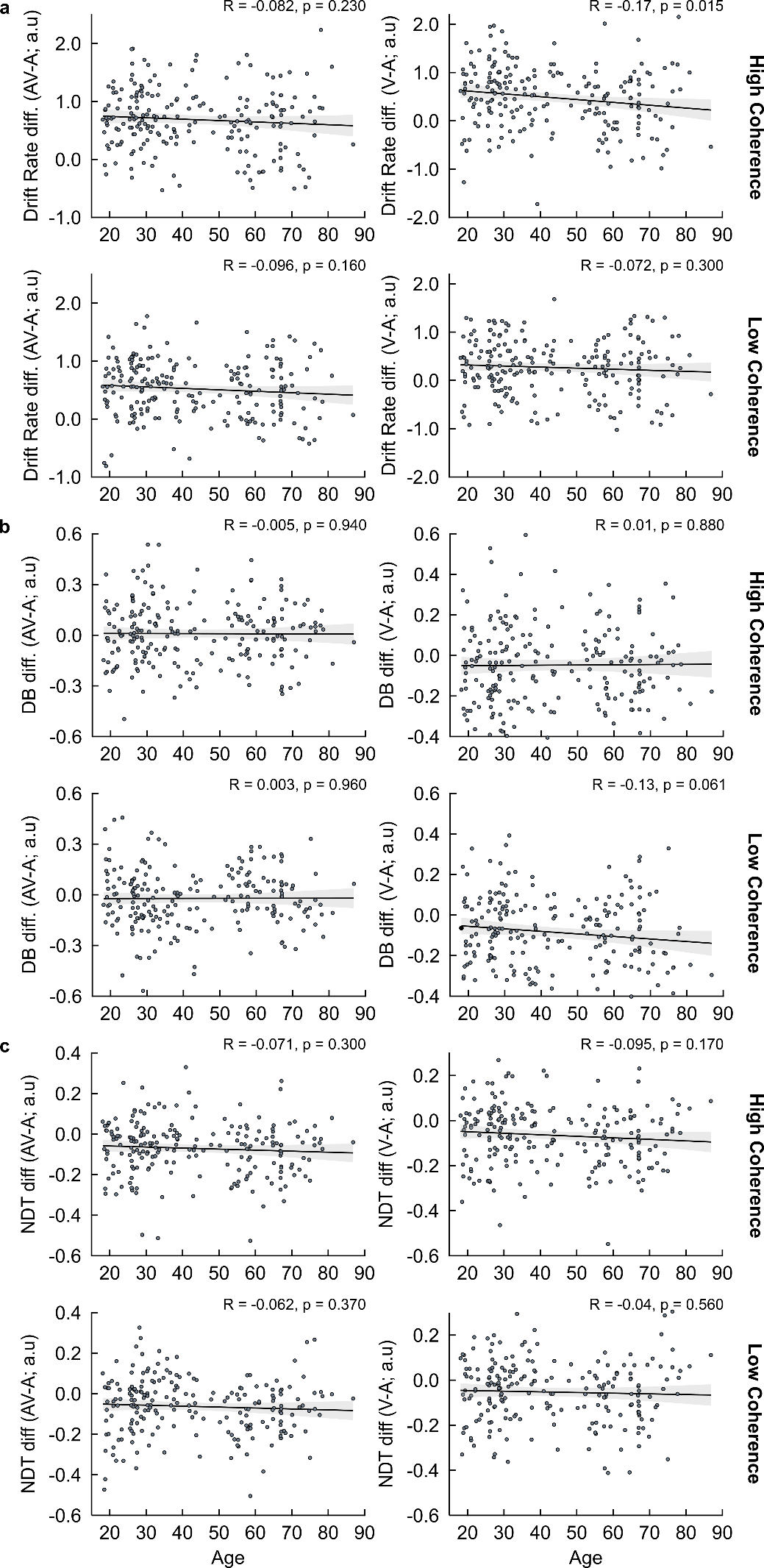


**Supplementary Figure 8: (A)V versus A HDDM Posterior Parameter Estimations - Performance Differences.** Individual Pearson’s correlations of chronological age with **a,** drift rate (δ), **b,** decision boundary (DB, θ), and **c,** non-decision time (NDT, τ) parameter estimate differences between AV versus V conditions (*left*) and V versus A conditions (*right*) across levels stimulus coherence (high/low coherence). Pearson Correlation Coefficients (R) and p-values are shown for each correlation. Shaded regions indicate 95% Confidence Intervals (CIs).
