# Supplementary material for: A drift diffusion model analysis of age-related impact on multisensory decision-making processes
Source: Sci Rep. 2024 Jun 28;14:14895. doi: 10.1038/s41598-024-65549-5 (PMC11213863; doi:10.1038/s41598-024-65549-5)
Supplement: Supplementary file 2 — Supplementary Tables. [file 41598_2024_65549_MOESM2_ESM.docx]

| **IV Conditional Dependencies** | | | **Bayesian Hypotheses Testing** | | | |
| --- | --- | --- | --- | --- | --- | --- |
| Sensory Condition | Stimulus Coherence | Age Group | Parameter | Hypothesis | Non-Overlap of Posterior Proportions | Logit-Odds Proportion |
| AV  versus  V | HC | OA | Drift Rate  (δ) | $\delta_{AV}> \delta_{V}$ | **0.996** | **5.538** |
|  | LC |  |  |  | **0.986** | **4.278** |
|  | HC | YA |  |  | 0.948 | 2.902 |
|  | LC |  |  |  | **0.997** | **6.570** |
|  | HC | OA | Decision Boundary  (θ) | $\theta_{AV}< \theta_{V}$  (*****$\theta_{AV}> \theta_{V}$) | 0.075 (*0.925) | -2.515 (*2.515) |
|  | LC |  |  |  | 0.001 (***0.999**) | -6.660 **(*6.660)** |
|  | HC | YA |  |  | 0.034 (***0.966**) | -3.355 **(*3.355)** |
|  | LC |  |  |  | 0.249 (*0.751) | -1.106 (*1.106) |
|  | HC | OA | Non-Decision Time  (τ) | $\tau_{AV}< \tau_{V}$ | 0.643 | 0.587 |
|  | LC |  |  |  | **0.986** | **4.278** |
|  | HC | YA |  |  | 0.674 | 0.725 |
|  | LC |  |  |  | 0.224 | -1.242 |
| AV  versus  A | HC | OA | Drift Rate  (δ) | $\delta_{AV}> \delta_{A}$ | **> 0.999** | **> 6.660** |
|  | LC |  |  |  | **> 0.999** | **> 6.660** |
|  | HC | YA |  |  | **> 0.999** | **> 6.660** |
|  | LC |  |  |  | **> 0.999** | **> 6.660** |
|  | HC | OA | Decision Boundary  (θ) | $\theta_{AV}< \theta_{A}$  (*$\theta_{AV}> \theta_{A}$) | 0.463 (*0.537) | -0.150 (*0.150) |
|  | LC |  |  |  | 0.865 (*0.135) | 1.855 (*-1.855) |
|  | HC | YA |  |  | 0.327 (*0.673) | -0.721 (*0.721) |
|  | LC |  |  |  | 0.569 (*0.431) | 0.277 (*-0.277) |
|  | HC | OA | Non-Decision Time  (τ) | $\tau_{AV}< \tau_{A}$ | 0.882 | 2.010 |
|  | LC |  |  |  | 0.799 | 1.379 |
|  | HC | YA |  |  | **0.992** | **4.877** |
|  | LC |  |  |  | **0.995** | **5.246** |
| V  versus  A | HC | OA | Drift Rate  (δ) | $\delta_{V}> \delta_{A}$ | **> 0.999** | **> 6.660** |
|  | LC |  |  |  | **0.966** | **3.343** |
|  | HC | YA |  |  | **> 0.999** | **> 6.660** |
|  | LC |  |  |  | **> 0.999** | **> 6.660** |
|  | HC | OA | Decision Boundary  (θ) | $\theta_{V}< \theta_{A}$  (*$\theta_{V}> \theta_{A}$) | 0.917 (*0.083) | 2.401 (*-2.401) |
|  | LC |  |  |  | **< 0.001 (* > 0.999)** | **< -6.660 (*> 6.660)** |
|  | HC | YA |  |  | 0.929 (*0.071) | 2.567 (*-2.567) |
|  | LC |  |  |  | 0.809 (0.191) | 1.440 (-1.440) |
|  | HC | OA | Non-Decision Time  (τ) | $\tau_{V}< \tau_{A}$ | 0.796 | 1.362 |
|  | LC |  |  |  | 0.087 | 2.346 |
|  | HC | YA |  |  | **0.978** | **3.782** |
|  | LC |  |  |  | **0.999** | **6.660** |
| **Conditional Dependencies** | | | **Bayesian Hypotheses Testing** | | | |
| Sensory Condition | Stimulus Coherence | Age Group | Parameter | Hypothesis | Non-Overlap of Posterior Proportions | Logit-Odds Proportion |
| V | HC | OA  versus  YA | Drift Rate  (δ) | $\delta_{YA}> \delta_{OA}$ | **> 0.999** | **> 6.660** |
|  | LC |  |  |  | **> 0.999** | **> 6.660** |
|  | HC | OA  versus  YA | Decision Boundary  (θ) | $\theta_{YA}< \theta_{OA}$  (*****$\theta_{YA}> \theta_{OA}$) | 0.879 (*0.121) | 1.980 (*-1.980) |
|  | LC |  |  |  | 0.229 (*0.771) | -1.212 (*1.212) |
|  | HC | OA  versus  YA | Non-Decision Time  (τ) | $\tau_{YA}< \tau_{OA}$ | 0.352 | -0.609 |
|  | LC |  |  |  | **0.979** | **3.846** |
| A | HC | OA  versus  YA | Drift Rate  (δ) | $\delta_{YA}> \delta_{OA}$ | **> 0.999** | **> 0.660** |
|  | LC |  |  |  | **0.996** | **5.488** |
|  | HC | OA  versus  YA | Decision Boundary  (θ) | $\theta_{YA}< \theta_{OA}$  (*****$\theta_{YA}> \theta_{OA}$) | 0.875 (*0.125) | 1.949 (*-1.949) |
|  | LC |  |  |  | **0.996 (*0.004)** | **5.488 (*-5.488)** |
|  | HC | OA  versus  YA | Non-Decision Time  (τ) | $\tau_{YA}< \tau_{OA}$ | 0.076 | -2.495 (*2.495) |
|  | LC |  |  |  | **0.006 (*0.994)** | **-5.158 (*5.158)** |
| AV | HC | OA  versus  YA | Drift Rate  (δ) | $\delta_{YA}> \delta_{OA}$ | **> 0.999** | **> 6.660** |
|  | LC |  |  |  | **> 0.999** | **> 6.660** |
|  | HC | OA  versus  YA | Decision Boundary  (θ) | $\theta_{YA}< \theta_{OA}$  (*****$\theta_{YA}> \theta_{OA}$) | 0.775 (*0.225) | 1.235 (*-1.235) |
|  | LC |  |  |  | **0.952 (*0.048)** | **2.997 (*-2.997)** |
|  | HC | OA  versus  YA | Non-Decision Time  (τ) | $\tau_{YA}< \tau_{OA}$ | 0.374 | -0.516 |
|  | LC |  |  |  | 0.156 | -1.686 |
| **IV Conditional Dependencies** | | | **Bayesian Hypotheses Testing** | | | |
| Sensory Condition | Stimulus Coherence | Age Group | Parameter | Hypothesis | Non-Overlap of Posterior Proportions | Logit-Odds Proportion |
| V | HC  versus  LC | OA | Drift Rate  (δ) | $\delta_{HC}> \delta_{LC}$ | **0.999** | **6.660** |
|  |  | YA |  |  | **> 0.999** | **> 6.660** |
|  | HC  versus  LC | OA | Decision Boundary  (θ) | $\theta_{HC}< \theta_{LC}$  (*****$\theta_{HC}> \theta_{LC}$) | **0.002 (*0.998)** | **-6.213 (*6.213)** |
|  |  | YA |  |  | 0.174 (*0.826) | 1.556 |
|  | HC  versus  LC | OA | Non-Decision Time  (τ) | $\tau_{HC}< \tau_{LC}$ | **0.958** | **3.126** |
|  |  | YA |  |  | 0.253 | -1.083 |
| A | HC  versus  LC | OA | Drift Rate  (δ) | $\delta_{HC}> \delta_{LC}$ | **0.957** | **3.091** |
|  |  | YA |  |  | **0.998** | **6.064** |
|  | HC  versus  LC | OA | Decision Boundary  (θ) | $\theta_{HC}< \theta_{LC}$  (*****$\theta_{HC}> \theta_{LC}$) | 0.471 (*0.529) | -0.114 (*0.114) |
|  |  | YA |  |  | 0.056 (*0.944) | -2.832 (*2.832) |
|  | HC  versus  LC | OA | Non-Decision Time  (τ) | $\tau_{HC}< \tau_{LC}$ | 0.313 | -0.788 |
|  |  | YA |  |  | 0.746 | 1.078 |
| AV | HC  versus  LC | OA | Drift Rate  (δ) | $\delta_{HC}> \delta_{LC}$ | **> 0.999** | **> 6.660** |
|  |  | YA |  |  | **> 0.999** | **> 6.660** |
|  | HC  versus  LC | OA | Decision Boundary  (θ) | $\theta_{HC}< \theta_{LC}$  (*****$\theta_{HC}> \theta_{LC}$) | 0.108 (*0.892) | -2.113 (2.113) |
|  |  | YA |  |  | **0.016 (*0.984)** | **-4.137 (*4.137)** |
|  | HC  versus  LC | OA | Non-Decision Time  (τ) | $\tau_{HC}< \tau_{LC}$ | 0.445 | -0.223 |
|  |  | YA |  |  | 0.708 | 0.885 |

**Supplementary Tables T1, T2, and T3: HDDM Hypothesis Testing Results.** HDDM hypothesis testing results for sensory condition, age group, and stimulus coherence respectively. Shaded Non-Overlap of Posterior Proportions and Logit-Odds Proportions cells indicate strongly predictive effects of corresponding hypotheses.
